# Improved resolution of 3-mercaptopropionate dioxygenase active site provided by ENDOR spectroscopy offers insight into catalytic mechanism

**DOI:** 10.1016/j.jbc.2024.105777

**Published:** 2024-02-21

**Authors:** Brad S. Pierce, Allison N. Schmittou, Nicholas J. York, Ryan P. Madigan, Paula F. Nino, Frank W. Foss, Molly M. Lockart

**Affiliations:** 1 Department of Chemistry & Biochemistry, University of Alabama, Tuscaloosa, Alabama, USA; 2 Department of Chemistry & Biochemistry, The University of Texas at Arlington, Arlington, Texas, USA; 3 Department of Chemistry and Biochemistry, Samford University, Homewood, Alabama, USA

**Keywords:** thiol dioxygenase, mononuclear nonheme iron, hydrogen bonding, sulfur-oxidation, pulsed EPR spectroscopy, ^1^H Mims ENDOR, computational, DFT

## Abstract

3-mercaptopropionate (3MPA) dioxygenase (MDO) is a mononuclear nonheme iron enzyme that catalyzes the O_2_-dependent oxidation of thiol-bearing substrates to yield the corresponding sulfinic acid. MDO is a member of the cysteine dioxygenase family of small molecule thiol dioxygenases and thus shares a conserved sequence of active site residues (Serine-155, Histidine-157, and Tyrosine-159), collectively referred to as the SHY-motif. It has been demonstrated that these amino acids directly interact with the mononuclear Fe-site, influencing steady-state catalysis, catalytic efficiency, O_2_-binding, and substrate coordination. However, the underlying mechanism by which this is accomplished is poorly understood. Here, pulsed electron paramagnetic resonance spectroscopy [^1^H Mims electron nuclear double resonance) spectroscopy] is applied to validate density functional theory computational models for the MDO Fe-site simultaneously coordinated by substrate and nitric oxide (NO), (3MPA/NO)-MDO. The enhanced resolution provided by electron nuclear double resonance spectroscopy allows for direct observation of Fe-bound substrate conformations and H-bond donation from Tyr159 to the Fe-bound NO ligand. Further inclusion of SHY-motif residues within the validated model reveals a distinct channel restricting movement of the Fe-bound NO-ligand. It has been argued that the iron-nitrosyl emulates the structure of potential Fe(III)-superoxide intermediates within the MDO catalytic cycle. While the merit of this assumption remains unconfirmed, the model reported here offers a framework to evaluate oxygen binding at the substrate-bound Fe-site and possible reaction mechanisms. It also underscores the significance of hydrogen bonding interactions within the enzymatic active site.

Abnormalities in sulfur metabolism have been correlated to oxidative stress and neurodegenerative disease (1, 2). Consequently, enzymes involved in sulfur oxidation are common targets for the development of novel treatments for cancer and inflammatory disease (3, 4, 5, 6). Thiol dioxygenases (TDOs), part of the mononuclear nonheme iron oxygenase family, play a role in sulfur oxidation reactions. These enzymes catalyze O_2_-dependent oxidation of thiol-bearing substrates to yield sulfinic acids. In mammals, cysteine dioxygenase (CDO) and cysteamine dioxygenase (ADO) are the only known TDOs (7, 8), catalyzing the oxidation of L-cysteine (CYS) and cysteamine (CA) to produce cysteine sulfinic acid and hypotaurine, respectively (9, 10, 11). Although CDO and ADO share structurally similar substrates, they demonstrate high substrate-specificity, displaying negligible cross-reactivity (7). Notably, variations in cellular expression and functioning of CDO and ADO have been linked to multiple human diseases, including cancer, neurodegenerative disorders, rheumatoid arthritis, and metabolic disorders (12, 13, 14, 15, 16, 17, 18, 19).

Beyond mammalian CDO and ADO, numerous TDOs have been identified across phylogenic domains. A noteworthy development in this field is the discovery of a subset of TDOs that exhibit N-terminal cysteinyl dioxygenase activity (20). These enzymes (referred to as NCOs) function as oxygen sensors by catalyzing the oxidation of protein N-terminal CYS-residues to initiate the degradation of proteins. For example, plant cysteine oxidases (PCOs) catalyze the formation of N-terminal cysteine sulfinate on the ethylene response factor VII (ERFVIIs) protein, thereby initiating N-end rule degradation (21, 22). Interestingly, it has been reported that mammalian ADO exhibits a comparable function in regulating regulators of G protein signaling (23). Thus, ADO functions as a dual-role enzyme, acting as both a small molecule TDO to produce hypotaurine from CA as well as an O_2_-dependent NCO regulating stability of specific proteins (20, 24, 25).

Sequence alignment of functionally and/or structurally characterized TDOs reveal considerable homology among TDOs that catalyze reactions with small organic substrates (CDO, MDO, and mercaptosuccinate dioxygenase). This group is referred to as “small molecule” TDOs (smTDOs) to differentiate them from NCOs (20). These enzymes belong to the Pfam-identified CDO_I family (PF05995) and share many conserved sequence motifs, including a fundamental conservation of the three His residues that bind to the mononuclear iron site. Additionally, three spatially adjacent residues (Ser153, His155, and Tyr157, *Rattus norvegicus* CDO numbering) are highly conserved and form a hydrogen bonding (H-bonding) network within the enzymatic active site. We have introduced the term “SHY-motif” to refer to the triad of Serine155, Histidine157, and Tyr159 residues collectively (26, 27). A comparison of smTDO sequences across phylogenic domains was used to generate the sequence logo presented in Figure 1*A* (https://efi.igb.illinois.edu/efi-est/) (28). While His- and Tyr-residues are conserved, some variation in the Ser-residue is observed. However, in these instances, a functionally equivalent residue is substituted [Thr (15%), Asn (4%), or His (2%)] such that the H-bonding network is maintained (13). This consensus among SHY residues led to the initial assumption that these amino acids comprise a “catalytic triad.” (13).

**Figure 1:**
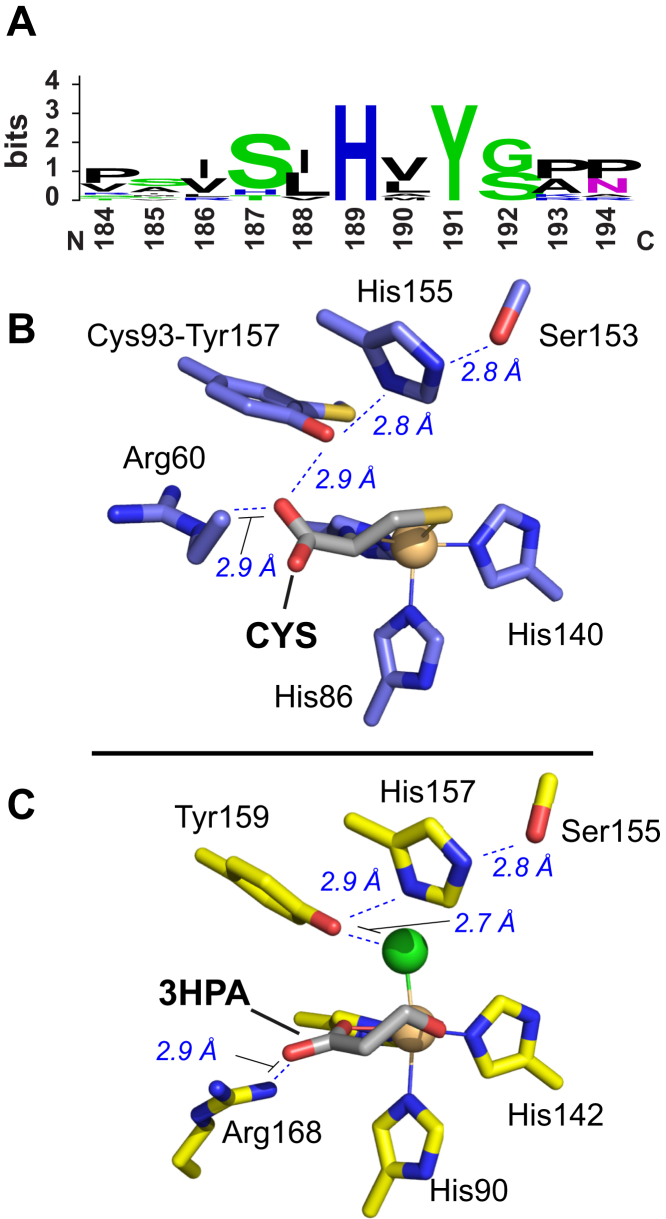
**Conserved SHY-motif within small molecule TDO active site.***A*, logo presentation of consensus SHY-motif among characterized small molecule TDOs. A comparison of X-ray crystal structures for (*B*) CYS-bound *Rattus norvegicus* CDO active site (PDB: 4IEV) and (*C*) 3HPA-bound *Azotobacter vinelandii* MDO (PDB: 6XB9). Of note, the CDO Cys92-Tyr157 cross-link is produced over hundreds of catalytic turnovers. Therefore, the purified enzyme is typically a mixture of uncross-linked and cross-linked forms. The XRD structure shown in (*B*) was obtained from crystals soaked with excess CYS (pH 8.0) under aerobic conditions to favor stoichiometric cross-link formation (29). 3HPA, 3-hydroxypropionic acid; CDO, cysteine dioxygenase; CYS, L-cysteine; MDO, 3-mercaptopropionate dioxygenase; PDB, Protein Data Bank; SHY, Ser-His-Tyr; TDO, thiol dioxygenases; XRD, X-Ray diffraction analysis.

Figure 1 illustrates a comparison of the active site structures for eukaryotic CDO and bacterial MDO (29, 30, 31, 32, 33). As members of the cupin superfamily (8, 34), smTDOs share a mostly invariant topology, with an overall RMSD of ∼1.2 Å [<0.3 Å among active site residues] (35). Notably, mammalian CDOs feature a unique posttranslational modification in which the terminal tyrosine (Tyr157) in the Ser-His-Tyr network is covalently cross-linked with an adjacent Cys-residue (Cys93) to form a Cys93-Tyr157 pair (C93-Y157) as shown in Figure 1 *B*. This posttranslational modification improves catalytic and coupling efficiency of the mammalian enzyme (29, 36, 37). By contrast, bacterial MDO retains an unmodified Tyr at this position.

Similar to other smTDOs (38), CDO displays bidentate substrate binding, where CYS coordinates to the Fe-site *via* a neutral amine and thiolate group trans to His140 and His88, respectively. Outer-sphere interactions with Tyr157 and Arg60 residues further contribute to CDO's high substrate specificity. Crystal structures for the MDO-3MPA complex are currently unavailable. The structure shown in Figure 1*C* depicts MDO in complex with a substrate analog and competitive inhibitor (3-hydroxypropionic acid [3HPA], Protein Data Bank [PDB] code: 6XB9) (39). Here, 3HPA is bound to iron by both its terminal hydroxyl group and carboxylate *trans* to His92 and His142, respectively. Also observed is an iron-coordinated chloride *trans* to His90 which interacts with the hydroxyl group of Tyr159 to form an ion-dipole interaction. This represents the putative dioxygen binding site and is designated the axial Fe-position to differentiate it from the equatorial substrate-binding sites (27, 39, 40). 3HPA differs from the native substrate by substitution of a single O-atom (hydroxyl *versus* thiol), and thus it is reasonable to assume an equivalent bidentate coordination of the native 3MPA substrate. Indeed, computational studies, validated by hyperfine sublevel correlation spectroscopy (HYSCORE) and ^57^Fe-Mössbauer confirm bidentate coordination in the (3MPA/NO)-MDO ternary complex (39).

Members of the NCO subset of enzymes belong to the PCO_ADO family (PF07847) and are separated from the aforementioned smTDOs both functionally and evolutionarily (41). The early divergence of these enzyme clusters is characteristic of functional diversification within the enzyme superfamily. While NCOs also exhibit absolute conservation of iron binding His-residues, considerable sequence deviation is observed relative to smTDOs within the active site. For instance, the SHY-motif present in smTDOs is absent in ADO and PCO. Instead, these residues are replaced by an (X-Asp-Leu) sequence, resulting in the placement of an anionic Asp192 residue 2.9 Å from the Fe-site (24). Therefore, despite catalyzing the same overall chemical reaction, smTDOs, and NCOs exhibit considerable deviations in substrate-specificity and active site architecture.

Perturbations within the smTDO SHY-motif exert substantial effects on catalysis and substrate binding. Among these, variants that break the H-bonding network are the most impactful. This is exemplified by the CDO H155A variant, which exhibits a two order of magnitude decrease in *k*_cat_ relative to WT enzyme (42). Conversely, the H-bonding network is retained in the H157N MDO variant, and therefore only a ∼20-fold reduction in *k*_cat_ is observed. This attenuated activity for H157N MDO is also associated with an increased oxygen K_M_-value and decreased coupling efficiency (26, 37, 43). Similarly, electron paramagnetic resonance (EPR) experiments using nitric oxide (NO) and cyanide as spectroscopic probes reveal decreased binding affinity for these ligands among SHY-variants (H157N and Y159F), suggesting their involvement in regulating dioxygen binding (26). Since the SHY-motif H-bonding network extends to the protein-solvent interface, these residues may function as a relay network to synchronize protonation of iron-oxo intermediates formed during catalysis. Indeed, solvent kinetic isotope effects, and proton-inventory experiments on CDO indicate a single rate-limiting proton following dioxygen binding (44). This may explain why a substitution of His with Asn (a nonionizable residue) is so impactful on MDO activity.

One aspect of the SHY-motif function that is poorly understood is its influence on the binding of substrates and exogenous ligands at the mononuclear iron site. Two specific experiments highlight this unusual behavior. First, the addition of NO to WT MDO preincubated with excess 3MPA results in a ternary complex of enzyme simultaneously coordinated by 3MPA and NO [(3MPA/NO)-MDO]. EPR signals observed for this complex are diagnostic of an iron-nitrosyl complex of intermediate spin (*S* = 3/2) (26, 33). Of note, the substitution of CYS for 3MPA yields an equivalent EPR signal along with a second (minor) feature indicative of a second CYS-binding conformation. This suggests that CYS and 3MPA produce an equivalent iron-nitrosyl complex. By extension, this means CYS also favors bidentate coordination at the Fe-site through carboxylate and thiolate functional groups in the WT enzyme. By contrast, equivalent experiments performed on the Y159F variant of MDO result in an atypical low-spin (*S* = 1/2) iron-nitrosyl complex (26). A nearly identical EPR signal is observed for the (CYS/NO)-CDO complex (45), suggesting that CYS-coordination within the Y159F MDO variant matches CDO; namely, bidentate coordination through neutral amine and thiolate groups. This implies that the removal of the Tyr159 hydroxyl group reverses the MDO CYS-binding specificity to favor thiolate/amine coordination rather than thiolate/carboxylate.

Second, it was recently demonstrated that H-bond donation by Tyr159 influences the position and number of coordinating cyanide ligands at the Fe(III)-MDO active site (27). These experiments suggest that intermolecular interactions with Tyr159 modulate ligand binding at the MDO Fe-site. It has been proposed that the charge of the substrate-bound Fe-site is a significant factor driving O_2_-reactivity in both CDO and MDO (46). Therefore, the observed influence of Tyr159 H-bond donation on anionic ligand binding at the MDO Fe-site implies that the SHY-motif may play a role in orchestrating the obligate ordered addition of organic substrate prior to dioxygen during catalysis.

Several high-resolution X-ray crystal structures are available for the substrate-bound CDO. However, the absence of comparable structures in other smTDOs hinders our understanding of SHY-motif function. In particular, it is difficult to extrapolate a mechanism by which Tyr159 orchestrates the coordination of axial and *equatorial* ligands without first establishing the orientation of the Tyr159 H-bond relative to the Fe-site. Attempts were made to extract this information from our previous ^1^H HYSCORE characterization of (3MPA/NO)-MDO. However, these efforts were hindered by poor dispersion of multiple overlapping weakly coupled protons. In the work presented here, we address this deficiency by applying ^1^H Mims electron nuclear double resonance (ENDOR) spectroscopy, which provides greater resolution of weakly coupled protons (<4 MHz) (47, 48). This analysis is aided by comparison of samples prepared from isotopically labeled substrates [2,2-^2^H-3MPA (d_2_-3MPA) and 2,2,3,3-^2^H-3MPA (d_4_-3MPA)] to those prepared from natural isotopic abundance 3MPA. These measurements provide greater precision in the placement of substrate protons, allowing for the differentiation of 3MPA-binding conformers. Additionally, difference spectra obtained from WT and H157N samples verify Tyr159 H-bond donation to the NO ligand bound to the axial Fe-positon. The spectroscopically validated models reported here provide the highest resolution model of the (3MPA/NO)-MDO active site to date.

## Results

### Computational modeling

The initial computational model for the (3MPA/NO)-MDO ternary complex was constructed using the coordinates obtained from the X-ray crystal structure of MDO in complex with the competitive inhibitor, 3HPA [PDB code: 6XB9] (39). In the structure, the chloride ligand occupying the putative dioxygen binding site *trans* to His90 was replaced by NO. Spectroscopic validation for this structure was obtained through pulsed EPR experiments (HYSCORE) performed on samples of enzyme simultaneously coordinated to 3MPA and NO. In addition, Mössbauer spectroscopic parameters observed for (3MPA/NO)-MDO were faithfully reproduced by computational models. Collectively, these studies support the assignment of bidentate 3MPA coordination at the Fe-site through thiolate and carboxylate functional groups.

Computational models and relaxed surface scan calculations suggested that the lowest energy conformation involved Tyr159 H-bond donation to the axially bound Fe-nitrosyl group. However, insufficient dispersion of weakly coupled protons was obtained from ^1^H HYSCORE spectroscopy to confirm this prediction. Consequently, no conclusions could be made regarding the orientation of Tyr159 H-bonding within the SHY motif.

### 3MPA conformers

**Figure 2:**
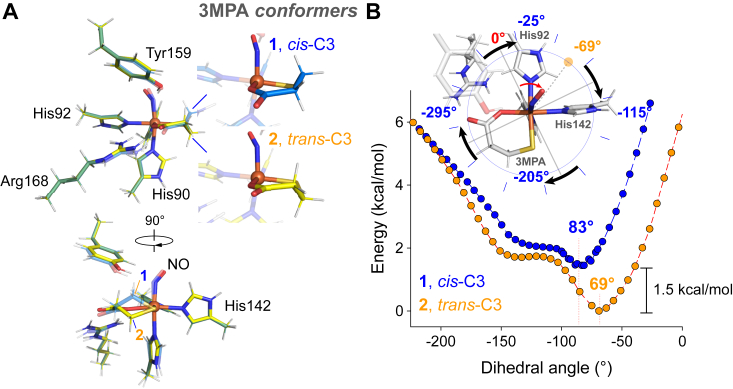
**Modeling of the (3MPA/NO)-MDO ternary complex.***A*, geometry optimized models of *cis*-C3 (1, *blue*) and *trans*-C3 (2, *yellow*) (3MPA/NO)-MDO sites. *B*, relaxed surface scan for each conformer illustrating the change in energy as a function of NO-dihedral angle. 3MPA, mercaptopropionic acid; MDO, 3-mercaptopropionate dioxygenase; NO, nitric oxide.

Bidentate coordination of 3MPA at the mononuclear Fe site forms a 6-membered ring. Much like cyclohexane, multiple conformations can be produced to minimize ring and torsional strain. In the original optimized structure shown in Figure 2*A*, C3 of 3MPA was staggered upward such that it was positioned above the plane of the ring. As this places both 3MPA C3 and the Fe-bound nitrosyl group on the same side of the ring, this conformer is referred to as *cis*-C3 (conformer ***1***). Similarly, a second conformer can be produced by staggering 3MPA C3 below the 6-membered ring placing it on the opposite side relative to the Fe-bound nitrosyl group (Fig. 2*A*, ***2***, *trans*-C3). Atomic coordinates for optimized *cis*-C3 and *trans*-C3 models are provided in Supporting Information, Section XI. Of note, the optimized energy for *cis*-C3 is +1.5 kcal/mol higher in energy relative to *trans*-C3. However, the *cis*-C3 conformer closely matches the crystal structure for the 3HPA-bound MDO complex (RMSD 0.417 Å) (35). It was therefore reasonable to assume that a similar *cis*-C3 conformer was adopted for the 3MPA-bound Fe-site. Significantly, as noted in Table S3, the relative positioning of 3MPA C2 and C3 axial and equatorial protons are divergent for the *cis*-C3 and *trans*-C3 conformers. This is expected to alter the observed dipolar couplings for each set of protons.

### Orientation of the Fe-bound nitrosyl ligand

Both optimized density functional theory (DFT) models predict an iron nitrosyl (Fe-N-O) bond angle [*cis*-C3 (141°) and *trans*-C3 (139°)] consistent with crystallographically characterized iron-nitrosyl clusters sites of intermediate spin (*S* = 3/2) (49, 50). The stick model shown in Figure 2 suggests that the Fe-bound NO ligand can freely rotate around the His90-Fe-N_NO_ axis. However, when shown with appropriate van der Waal radii (Fig. S6), it is apparent that considerable steric bulk within the first- and outer-coordination sphere of the MDO Fe-site limits rotation of the nitrosyl ligand. Moreover, H-bond donation from Tyr159 is expected to further promote a homogeneous NO-binding position. However, placement of 3MPA C3 above (*cis*-C3) or below (*trans*-C3) the plane of the 6-membered ring will alter the steric bulk within the active site thereby altering the placement of the Fe-bound nitrosyl ligand. To illustrate this point, we define a NO-dihedral angle by C_δ2_, N_ε2_ atoms of His90, and the N- and O-atoms of the NO ligand. In multiple optimizations of *cis*-C3, the final NO-dihedral angle converges to −84 ± 2° regardless of the initial angle posed. Similarly, optimizations of *trans*-C3 reproducibly favor a dihedral angle of −70 ± 2°. The exact positioning of Fe-N-O atoms (relative to coupled protons) will significantly influence the reliability of point dipole approximations used to simulate ^1^H Mims ENDOR data (51, 52, 53). Therefore, to verify that these dihedral angles represent the most likely placement of NO ligands, relaxed surface scan calculations were performed for each conformer (54, 55, 56). Figure 2*B* presents the calculated energy surface for each conformer upon rotation of the NO ligand from −240° to +15°. Beyond these angles, optimized structures did not converge, suggesting energetically unfavorable structural rearrangements. Significantly, the energy surface for conformers **1** and **2** exhibit a minimum at dihedral angles −83° and −69°, respectively. This supports the argument that that steric bulk and Tyr159 H-bonding produce a uniform NO-binding geometry. This also validates the use of a point dipole approximation to estimate proton dipolar couplings observed in ^1^H Mims ENDOR studies.

### Pulsed EPR measurements

In the initial ^1^H HYSCORE analysis of (3MPA/NO)-MDO, field-dependent simulations were calculated assuming contributions of eight nearby protons, each with an individual hyperfine term and set of angles relating the Fe-^1^H vector and the magnetic axis system (39). Simulations calculated from the structural parameters obtained from optimized models reproduced the experimental ^1^H HYSCORE spectra. However, due to similarities in the dipolar coupling or nearby protons, many of these resonances overlap and cannot be unambiguously assigned. Thus, despite its ability to verify the overall conformation of 3MPA binding at the MDO Fe-site, this approach lacked the resolution to determine the exact positioning of individual protons relative to the iron center.

To address this deficiency, pulsed ENDOR experiments were performed to resolve weakly coupled protons. ENDOR is complementary to HYSCORE and can be used to obtain a simplified slice of field-dependent nuclear couplings. ENDOR is also better suited to resolve weakly coupled protons (<4 MHz) (47, 48).

In these experiments, ^1^H Mims ENDOR data were collected for samples of WT (3MPA/NO)-MDO prepared from naturally isotopically abundant substrate. Difference spectra were then obtained by subtraction of data obtained with equivalent samples prepared from 3MPA selectively ^2^H-labeled at C2 (d_2_-3MPA) and C2 and C3 (d_4_-3MPA).

**Figure 3:**
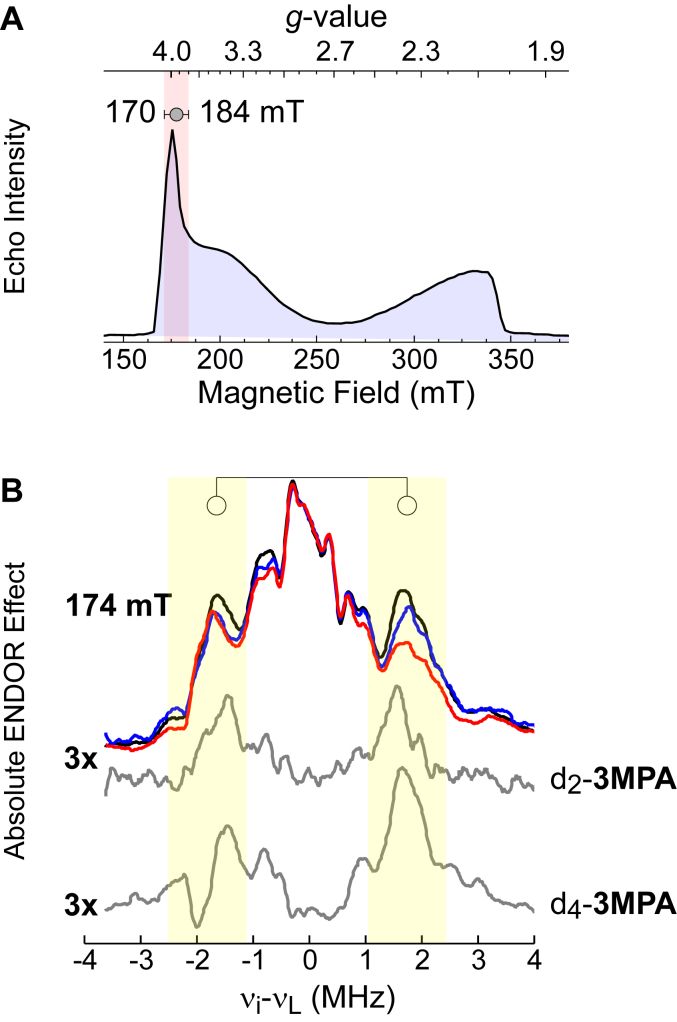
**Pulsed EPR characterization of (3MPA/NO)-MDO.***A*, X-Band two-pulse field-swept echo detected EPR spectra of (3MPA/NO)-MDO collected at 5 K. The iron-nitrosyl (*S* = 3/2) site exhibits near axial symmetry with *g*-values of 4, 4, and 2. The highlighted region near *g* ∼ 4 represents the magnetic field range (170–184 mT) where ^1^H Mims ENDOR spectra were collected. *B*, ^1^H Mims ENDOR spectrum for (3MPA/NO)-MDO at 174 mT with natural isotopic abundance 3MPA (*black*), d_2_-3MPA (*blue*) and d_4_-3MPA (*red*). Spectral regions showing the greatest deviation from natural isotopic abundance are indicated by the *circles*. Difference spectra obtained from subtracting isotopically enriched samples (d_2_-3MPA and d_4_-3MPA) from natural isotopic abundance samples are shown in *gray*. For best peak visibility the intensity of the resulting difference spectra was multiplied 3-fold. 3MPA, mercaptopropionic acid; ENDOR, electron nuclear double resonance; EPR, electron paramagnetic resonance; MDO, 3-mercaptopropionate dioxygenase; NO, nitric oxide.

Shown in Figure 3*A* is the pulsed EPR spectra observed for the (3MPA/NO)-MDO (*S* = 3/2) iron-nitrosyl species. Figure 3*B* (black trace) shows the ^1^H Mims ENDOR spectrum for (3MPA/NO)-MDO using natural isotopic abundant substrate collected at 174 mT. As previously reported, the greatest ^1^H intensities are observed within field range of 170 to 184 mT (26, 39). Overlaid for comparison are the ENDOR spectra obtained from samples prepared with d_2_-3MPA (blue) and d_4_-3MPA (red). Significantly, both isotopically labeled samples reveal decreased intensity reflecting a loss of protons. By simulating difference spectra (Fig. 3, gray) obtained across multiple field positions, the position and orientation of selected protons can be determined relative to the iron-nitrosyl.

**Figure 4:**
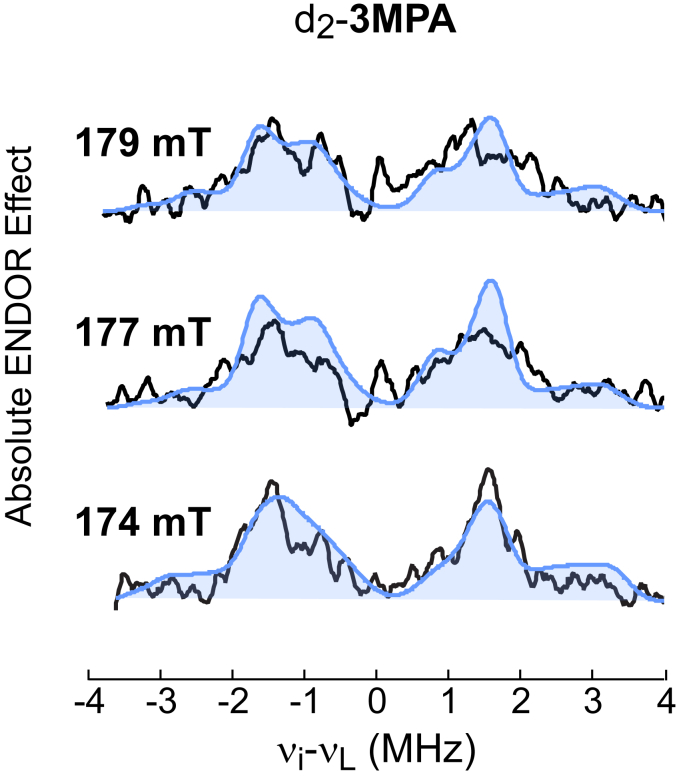
**(3MPA/NO)-MDO [**^**1**^**H-**^**2**^**H] difference ENDOR spectra obtained from d**_**2**_**-3MPA.** Difference spectra (*black traces*) were obtained by subtracting ENDOR spectra obtained with d_2_-3MPA from samples prepared with natural isotopic abundance. Field-dependent simulations for 3MPA C2-protons (*blue trace*) are overlaid on difference spectra for comparison. Distance and orientations of C2 protons were taken from DFT-optimized models. Since the position of C2 protons is not significantly altered in *cis*-C3 and *trans*-C3 isomerization, simulations generated from either optimized conformer (1 or 2) fit equally well. 3MPA, mercaptopropionic acid; DFT, density functional theory; ENDOR, electron nuclear double resonance; MDO, 3-mercaptopropionate dioxygenase.

Figure 4 shows the difference spectra for samples prepared with d_2_-3MPA across three representative fields. For brevity, a full presentation of all six field-dependent difference spectra and corresponding simulations is presented in (Fig. S7). The observed peaks can be attributed to the C2 protons on 3MPA, and thus their position can be used to validate computational models generated for the (3MPA/NO)-MDO active site. The placement of C2 protons is not significantly altered in *cis*-C3 and *trans*-C3 conformational models. Therefore, reasonable simulations (Fig. 4, filled blue trace) are obtained using either model.

**Figure 5:**
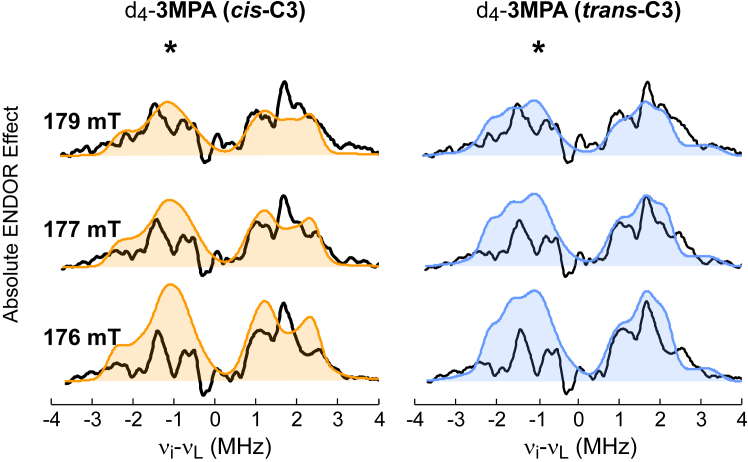
**(3MPA/NO)-MDO [**^**1**^**H-**^**2**^**H] difference ENDOR spectra obtained from d**_**4**_**-3MPA.** Difference spectra (*black traces*) were obtained by subtracting ENDOR spectra obtained with d_4_-3MPA from samples prepared with natural isotopic abundance. Field-dependent simulations for 3MPA C2 and C3-protons are overlaid on difference spectra for comparison. Distance and orientations of for C2 and C3 protons were taken from DFT-optimized models (1, *cis*-C3, *orange*) and (2, *trans*-C3, *blue*). The region of the spectra suppressed by implicit TRIPLE effect marked (∗) for clarity. 3MPA, mercaptopropionic acid; DFT, density functional theory; ENDOR, electron nuclear double resonance; MDO, 3-mercaptopropionate dioxygenase.

Figure 5 presents three representative difference spectra obtained with d_4_-3MPA at selected magnetic field positions (Fig. S8 shows all six field-dependent difference spectra collected with overlaid simulations). Before discussing simulations for these spectra, some additional context is necessary. At some field positions, the peak intensity arising from nuclear transitions within one spin manifold is suppressed relative to transitions within the other spin manifold. This results in an asymmetry of peak intensity observed on one side of the proton Larmor frequency when compared to the other. This phenomenon has been described previously and is referred to as the “implicit TRIPLE effect” (57, 58, 59). This can be observed in the d_4_-3MPA difference spectra and is indicated by (∗) in Figure 5. This effect is often observed in the presence of strong ^14^N modulation from nearby N-atoms and thus is not unexpected for signals from the 3-His coordinated MDO. In part, the amount of suppression depends on the delay times used between microwave pulses in the measurement. Under optimal measurement conditions for these samples, the peak suppression is most prominent in the lower-frequency side of the difference spectra. Therefore, the intensity of peaks on the higher-frequency side of the spectra more accurately represents the 3MPA protons.

On the left side of Figure 5, simulations (filled orange trace) generated from the couplings and orientations predicted from the *cis*-C3 (conformer **1**) are overlaid on the experimental difference spectra. For comparison, an equivalent set of simulations (filled blue trace) generated from the *trans*-C3 (conformer **2**) model is presented on the right side of Figure 5. As noted above, the higher frequency peaks within the difference ENDOR spectra are not influenced by the implicit TRIPLE effect. Therefore, these peaks were used to evaluate the quality of spectroscopic simulations. Significantly, simulations generated from the *trans*-C3 conformer more faithfully reproduce the number of peaks and their relative intensity across all field positions. Based on this result we conclude that conformer ***2*** (*trans*-C3) more accurately represents the structure of the (3MPA/NO)-MDO complex.

As an additional validation, the computational model for conformer **2** (*trans*-C3) was used to resimulate our previous ^1^H HYSCORE results to ensure that conformational changes did not introduce any unanticipated changes in global fits. As shown in Figs. S9 and S10, ^1^H HYSCORE simulations generated based on the new, *trans*-C3 3MPA conformation are superimposable with those generated from the initial *cis*-C3 model (conformer **1**). This demonstrates that ^1^H HYSCORE spectroscopy cannot distinguish between these 3MPA conformations in the presence of overlapping protons. It also highlights the utility of ENDOR spectroscopy for the positional assignment of substrate protons; thereby, allowing for discrimination of *cis*-C3 and *trans*-C3 conformers.

**Figure 6:**
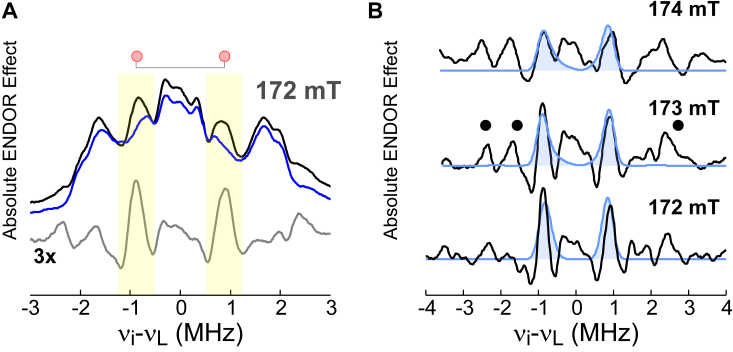
**(3MPA/NO)-MDO [WT-H157N] difference ENDOR spectra.***A*, ^1^H Mims ENDOR spectrum for WT (*black*) and H157N (*blue*) (3MPA/NO)-MDO at 172 mT. Spectra features showing the greatest are indicated by the *red circles*. *B*, difference spectra obtained by subtracting H157N from WT data at selected fields. Simulations (*blue shaded*) include the distances and orientations of the Try159-OH proton taken from the DFT-optimized model of (*trans*-C3-3MPA/NO)-bound MDO. The features indicated by (•) are attributed to a slight rotation of Fe-coordinated His residues as described in Fig. S7. 3MPA, mercaptopropionic acid; DFT, density functional theory; ENDOR, electron nuclear double resonance; MDO, 3-mercaptopropionate dioxygenase.

WT and H157N variant (3MPA/NO)-MDO have been extensively characterized by X-band CW EPR and Mössbauer spectroscopy. While this work is discussed elsewhere (26), representative CW EPR spectra obtained for WT and H157N MDO are presented in Fig. S11. Similar to the procedure outlined above, ENDOR spectra were collected for samples of (3MPA/NO)-MDO prepared from the H157N variant [(3MPA/NO)-H157N]. By subtracting these results from those obtained for WT (3MPA/NO)-MDO samples, difference spectra were obtained which isolate structural changes introduced by substitution of this single active site residue. Figure 6*A* presents the observed ^1^H Mims ENDOR spectrum for the WT (black trace) enzyme at 172 mT. At the same field, the observed ENDOR spectra for H157N samples (blue trace) are overlaid for comparison. The highlighted region reveals the absence of protons within samples of H157N that result in the difference spectra shown in gray.

At lower magnetic fields, the most obvious difference observed between WT and H157N is the absence of a pair of sharp peaks centered across the proton nuclear Larmor frequency. As this peak exhibits positive intensity, it must represent a ^1^H present in the WT enzyme that is absent in samples prepared from the H157N MDO variant. The weak coupling for this ^1^H matches (*T* = 0.94 MHz) what is expected for the outer-sphere Tyr159 phenol proton (39). Therefore, simulations were generated for this proton based on the optimized *trans*-C3 model. As shown in Figure 6*B*, simulations overlaid on H157N difference spectra perfectly match the observed peak across all field positions. A presentation of data collected across six magnetic fields and corresponding simulations is provided in Fig. S12. The loss of this proton in the H157N difference spectra suggests that the orientation of Tyr159 H-bond donation is shifted, moving the proton away from the iron-nitrosyl. To probe how this H-bond reorientation influences catalysis, temperature dependent kinetic measurements were performed on WT MDO and the H157N variant. These results are presented in Supporting Information, Section X, Fig. S14.

At higher magnetic fields, the intensity of the Try159-OH proton peaks decreases, and other features (indicated by • in Fig. 6*B*) within the difference spectra become more apparent. The increased splitting of these features indicated they originate from more strongly coupled protons, positioned closer to the iron-nitrosyl. Of note, these features appear derivative in nature, with both positive and negative lobes. This is indicative of a shift in proton hyperfine couplings or orientations rather than a loss of protons between WT and H157N spectra. Crucially, these features do not correspond to protons on 3MPA; instead, they have hyperfine couplings similar to protons on the Fe-coordinated histidine residues.

For Nε_2_-coordinated His-residues, the protons associated with [ε_1_ and δ_2_]-carbons have relatively large dipolar couplings ranging from 1.3 to 2.8 MHz. As shown in Supporting Information, Section IX, rotation along the (His)Nε_2_-Fe axis significantly alters the relative positioning of His142 and His92 protons relative to the magnetic coordinate system. Consequently, the rotation of these His-residues would be accompanied by a predictable shift in the observed ENDOR spectrum. By contrast, His90 protons are symmetrically positioned about the magnetic axis. Therefore, their coupling is largely independent of rotation and not reflected in the observed ^1^H ENDOR spectrum. Fig. S13 presents the dipolar coupling for protons on His142, His92, and His90 as a function of rotational angle.

To corroborate the assignment of these features, ENDOR simulations were calculated for different orientations of the closest protons on Fe-coordinated His residues. To best approximate a rotation of the H157N variant histidine residue relative to those in the WT enzyme, difference simulations were calculated by subtracting the H157N simulation from the WT. As illustrated in Fig. S13, many of these derivative features can be reproduced by difference simulations (red traces) across all field positions assuming a −15° (counter-clockwise) rotation of H157N His142 relative to WT (3MPA/NO)-MDO. Likewise, simulated difference spectra were considered for H157N His92 rotation. However, all of these features could not be matched by rotation of His142 or His92 alone. As stated above, the magnitude of His90 proton couplings is not appreciably influenced by rotation, and thus they cannot contribute to the observed difference spectra. We therefore speculate that the features shown in Figure 6 (•) result from a combination of His92 and His142 rotation. However, given the numerous combinations of His92 and His142 rotations (clockwise and counter-clockwise), it is not possible to validate the “uniqueness” of spectroscopic simulations. Regardless, His-rotation is the most plausible assignment for these features.

## Discussion

### Influence of Tyr159 H-bond donation

The mechanism for assembling the O_2_-reactive 3MPA:MDO complex is limited by the absence of high-resolution crystallographic information for this enzyme. Consequently, nearly all information regarding intermolecular interactions connecting the SHY-motif to the enzymatic Fe site is circumstantial. Comparison of ^1^H Mims ENDOR spectra obtained with isotopically abundant substrate with data obtained using selectively ^2^H-labeled 3MPA provides the relative positioning of protons in the active site. This information was used to validate structural models to determine substrate-binding geometry, denticity, and conformation. Similarly, a comparison of samples prepared from WT and H157N MDO variant allowed for the direct detection of outer-sphere Tyr159 H-bond donation to the axial Fe-nitrosyl ligand. As iron-nitrosyl complexes are often considered mimics for Fe-peroxo and Fe-superoxo intermediates, the structural properties of the (3MPA/NO)-MDO site are discussed, along with potential implications for proposed intermediates in the catalytic cycle of MDO.

Interactions between Tyr159 and coordinated ligands at the mononuclear nonheme iron site have been indicated by multiple spectroscopic, computational, and kinetic studies (26, 27). In one study involving the catalytically inert Fe(III)-MDO, it was demonstrated that cyanide could be used as a spectroscopic probe to observed Tyr159 H-bond donation within the WT enzyme to the axial Fe-position (27). Comparison to H157N MDO revealed diagnostic shifts in the low-spin ferric *g*-values observed by EPR and UV-visible absorption features attributed to the Fe(III)-S ligand-to-metal charge transfer (LMCT). These experimental parameters were faithfully reproduced by computational methods assuming reorientation of Tyr159 H-bond to favor donation to the δ_1_-O-atom of the Asn-amide (H157N), rather than Fe-bound cyanide. Notably, the Fe(III)-S LMCT is blue-shifted by 560 cm^−1^ (6.7 kJ‧mol^−1^) in the absence of Tyr159 H-bond donation.

The catalytic impact of Tyr159 H-bond reorientation within the functional Fe(II)-MDO enzyme is evident from the shift in the Tyr159 pK_a_-value (+0.7 pH units) when comparing the pH-dependent steady-state kinetic profile of WT and H157N MDO variant (26, 33). However, this insight gains further significance when considering the temperature dependence of MDO-catalyzed reactions (Supporting Information, Section X, Fig. S14). Under conditions of saturating oxygen and 3MPA, values for the WT MDO enthalpy (ΔH^‡^) and entropy (ΔS^‡^) of activation were measured at 34 ± 2 kJ‧mol^−1^ and −125 ± 9 J‧mol^−1^‧K^−1^, respectively. These values are similar to what has previously been reported for CDO (33). When compared to the values obtained for the H157N MDO variant [ΔH^‡^ = 41 ± 2 kJ‧mol^−1^ and ΔS^‡^ = −117 ± 8 J‧mol^−1^‧K^−1^], the influence of Tyr159 H-bond donation on transition state theory parameters can be isolated. Since kinetic measurements were made under saturating conditions, this cannot be attributed to changes in substrate-binding affinity. Instead, we argue that this shift is attributed to the reorientation of Tyr159 H-bond donation, which is confirmed by ENDOR spectroscopy. Remarkably, this 7 kJ‧mol^−1^ difference in ΔH^‡^ for WT and H157N variant closely aligns with the magnitude of blue-shifted Fe-LMCT (6.7 kJ‧mol^−1^) observed in the catalytically inactive Fe(III)-MDO (27). This suggests that the strength of Tyr159 H-bond donation is not significantly influenced by the charge and/or oxidation state of the Fe-site.

### Mechanistic considerations

**Figure 7:**
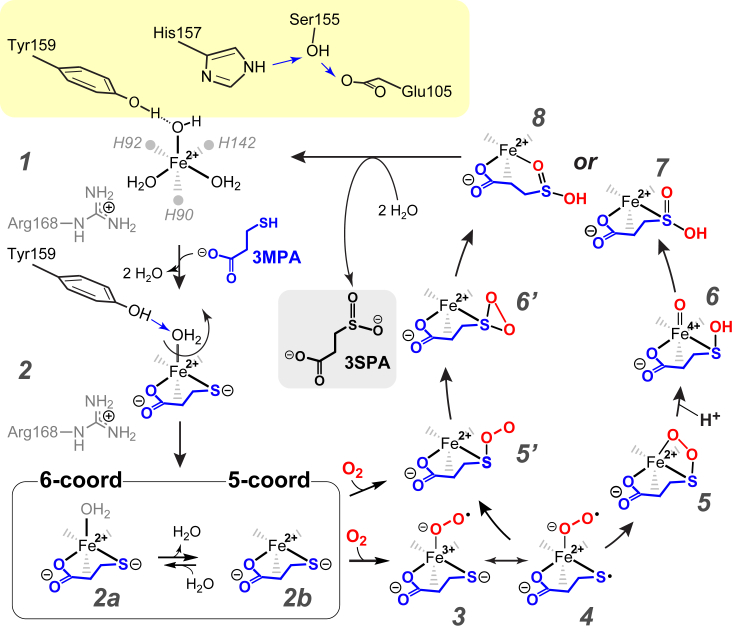
**Summary of proposed MDO reaction mechanism(s).** Shown in *yellow* is the H-bonding orientation of the conserved Ser-His-Tyr (SHY-motif) before substrate binding.

The crystal structure for a homologous MDO cloned from *Pseudomonas aeruginosa* [PDB code: 4TLF] reveals significant differences in the Fe-O bond distance of equatorial (2.6–2.7 Å) *versus* axially coordinated (2.3 Å) solvent waters (30). Therefore, in the “resting” (substrate-free) enzyme, the shorter bond distance of the axial solvent suggests this ligand is likely a hydroxide rather than a coordinated water. Energy surface calculations for the 3MPA-bound Fe-site indicate that Tyr159 H-bond donation to an axially coordinated anion (chloride and hydroxide) is lower in energy as compared to Tyr159 H-bond donation to His157 (Fig. S15, ***A*** and *B*) (39). Based on these observations, we propose the structure shown in Figure 7 (step 1) for the resting enzyme.

A single rate-limiting protonation step was identified by proton inventory and solvent kinetic isotope effects within the MDO *k*_cat_/K_M_-pL profile (32). This observation indicates that nonchemical events in the kinetic mechanism are rate-limiting. Since viscosity studies rule out rate-limiting product release, ionization of 3MPA upon coordination to iron is the most likely limiting process. Given the high pK_a_ of the 3MPA-thiol group, (pK_a_ = 10.8) (60), it is expected that formation of the deprotonated substrate dianion (3MPA^**2**−^) is rate-limiting at physiological pH. However, coordination of the 3MPA-thiol to the Lewis acidic Fe(II)-site likely decreases the thiol pK_a_ substantially (61, 62). It is also possible that the hydroxide bound to the axial Fe-position may function as a general base to facilitate this process as illustrated in Figure 7 (step **2**). The resulting enzyme-substrate complex then be a neutral 6-coordinate Fe-site with water coordinated at the axial position.

DFT optimized models of the 3MPA-bound Fe-site with axial bound water suggest that Tyr159 H-bond donation promotes disassociation of the *aqua* ligand resulting in a 5-coordinate complex (Fig. S14*C*). This model is supported by Mössbauer experiments on 3MPA-bound MDO in which Δ*E*_Q_-values observed are more consistent with five-coordinate model complexes of nearly equivalent first coordination sphere (63). We speculate that Tyr159 H-bond donation triggers the loss of the axially coordinated water as indicated by the equilibrium shown in Figure 7 (steps 2a ↔ 2b). Significantly, this substrate-gated decrease in iron coordination number has been observed spectroscopically for other mononuclear nonheme iron enzymes and provides a potential explanation for the obligate ordered addition of organic substrate before dioxygen (33, 63, 64).

It has been postulated that binding of molecular oxygen to the 5-coordinate 3MPA-bound Fe(II)-site (2b) yields a short-lived Fe(III)-superoxo species (Fig. 7, step ***3***). The transient radical character on the 3MPA S-atom (3 ↔ 4) could then allow for spin-allowed recombination of Fe(III)-bound superoxide and thiol-radical to yield a cyclic 4-membered Fe-O-O-S ring structure (5). The addition of a proton is expected to facilitate heterolytic cleavage of the O-O bond, resulting in formation of a transient Fe(IV)=O species (6) (65). Final rebound of the oxidizing Fe(IV)=O intermediate on the Fe-bound sulfenyl group regenerates the ferrous iron resting state of MDO in complex with the 3-sulfinopropionic acid product (7 or 8). Notably, this mechanism predicts the sequential addition of each O-atom to the organic substrate.

While no intermediates have been reported for MDO, independent investigations with CDO provide the most compelling support for an oxygen-activating thiol-dioxygenase mechanism. First, the catalytic cycle of mammalian CDO can be “primed” by one electron through chemical oxidation to produce CDO with ferric iron in the active site (66). Chemical-rescue experiments performed by the addition of superoxide to the substrate-bound Fe(III)-CDO yields a transient intermediate, which is kinetically matched to the formation of cysteine sulfinic acid. Characterization of this intermediate by UV-visible and parallel mode EPR spectroscopy is consistent with the formation of an oxidized iron-oxo species tentatively assigned as a CYS-bound Fe(III)-superoxide (analogous to Fig. 7, step 3) (66). Second, a short-lived (<20 ms) transient LMCT feature was observed by stopped-flow UV-visible spectroscopy upon rapid mixing of the substrate-bound CDO with saturating oxygen (31). Supporting time-dependent DFT-computational modeling of the transient UV-visible absorption spectra was reported which predict that this intermediate has a cyclic [Fe-O-O-S-CYS]-structure with a quintet (*S* = 2) spin-state (Fig. 7, step 5). Unfortunately, attempts to trap this intermediate and verify this assignment by rapid-freeze quench Mössbauer spectroscopy have been unsuccessful.

George *et al.* reported a potential-energy landscape for intermediates within the MDO catalytic cycle (63). Of note, the model for the putative Fe(III)-superoxide intermediate (Fig. 7, step 3) placed the superoxide distal O-atom directly above the substrate S-atom. This would correspond to a dihedral angle of −205 ± 15° in the coordinate system shown in Figure 2*B*. However, similar positioning of the distal O-atom for the iron-nitrosyl structure described in this work is incompatible with ENDOR simulations. In particular, couplings from the axial proton located at C2 of 3MPA (H85) and the Tyr159 phenol proton (H55) are significantly altered upon rotation of the Fe-bound nitrosyl. The van der Waal space fill (3MPA/NO)-MDO structure shown in Figure S6 also suggests that the iron-nitrosyl is constrained within a channel separating His142 and Tyr159. This is supported by the relaxed surface scans (Fig. 2*B*) which indicates that this orientation is optimal for maintaining Tyr159 H-bonding interactions and that the rotation of the iron-nitrosyl ligand is energetically unfavorable. This places the distal O-atom of the iron-nitrosyl 4.3 Å distant from the 3MPA S-atom. If we assume that the putative Fe(III)-superoxide intermediate adopts a similar binding conformation as the iron-nitrosyl, the distal O-atom would need to rotate clockwise by 136° to react with the 3MPA S-atom. As this rotation is energetically uphill, a direct attack of the superoxide proximal O-atom would seem more favorable. This suggests that either (3MPA/NO)-MDO is a poor structural mimic for the putative iron(III)-superoxide intermediate, or the proposed sequentia*l* oxygenation mechanism requires reconsideration.

An alternative mechanism was proposed by Karplus *et al.* based on a putative Fe(II)-bound persulfenate intermediate observed in the X-ray crystal structures for CYS-bound CDO [PDB code: 3ELN] (29, 67). In this mechanism, direct attack of the Fe(II)-thiolate by dioxygen is facilitated by the Lewis acidity of the Fe-site. Starting from 2b, dioxygen can either bind transiently at the Fe-site to yield the resonance structures (3 ↔ 4) or bypass these steps entirely by the concerted addition of dioxygen to the Fe-bound thiolate (5′). The addition of the distal O-atom to the S-atom of the Fe-bound persulfenate yields a transient thiadioxirane ring (6′). Ultimately, 3-sulfinopropionic acid is generated upon heterolytic cleavage of the O-O bond, yielding the enzyme-product complex (7 or 8).

Support for this mechanism can be taken from the crystal structure of the (CYS/NO)-CDO ternary complex [PDB code 6N43] reported by Li *et al.* (68) Consistent with the aforementioned putative persulfenate bond CDO structure, a shorter distance was observed between the substrate CYS sulfur and the proximal N-atom of the Fe-bound NO. This finding led to the conclusion that a concerted addition of dioxygen would be preferred over a sequential (68). By this reasoning, a similar argument can be made from the structure of the (3MPA/NO)-MDO ternary complex reported here. However, potential energy landscape calculations reported by Kumar *et al.* suggests the energy barrier for this mechanism is prohibitive (69). Consequently, the relevance of the Fe-bound persulfenate structure and the overall oxygenation mechanism for smTDOs remains a matter of some debate (29, 67, 70).

## Experimental procedures

### Enzyme expression and purification

Recombinant MDO cloned from *Azotobacter vinelandii* was expressed and purified as previously described (32, 33). In brief, the expression vector was transformed into chemically competent BL21(DE3) *Escherichia coli* and grown overnight at 37 °C on a 100 μg/L ampicillin LB-agar plate. A single colony was selected for growth in liquid LB media before inoculation of the 10-L BF-110 fermenter (New Brunswick Scientific) at 37 °C. Cell growth was monitored by absorbance at 600 nm (*A*_600_). When the *A*_600_ reached a value of ∼4, expression was induced by adding 1.0 mM IPTG, 20 μM ferrous ammonium sulfate, and 20 g casamino acids. Expression proceeded at 25 °C for 4 h. Cells were then harvested and pelleted by centrifugation at 5000 rpm (Beckman Coulter Avanti J-E, JA 9.1 rotor). The pelleted cell paste was frozen and stored at −80 °C.

In a typical purification, ∼20 g frozen cell paste was added to 20 mM 4-(2-hydroxyethyl)-1-piperazineethanesulfonic acid (Hepes), 50 mM NaCl, pH 8.0. Commercially available lysozyme, ribonuclease, and deoxyribonuclease (Sigma-Aldrich) were added for a final concentration of 10 μg/ml each and stirred slowly on ice for ∼20 min before pulse sonication (Bronson Digital 250/450). The sonicated mixture was centrifuged at 48,000*g* for 1 h at 4 °C (JA-20 rotor). The supernatant was then loaded onto a diethylaminoethyl cellulose Sepharose fast flow anion exchange column (GE Life Sciences #17070901) preequilibrated with 20 mM Hepes, 50 mM NaCl, pH 8.0. One column volume of 20 mM Hepes, 50 mM NaCl, pH 8.0 was used to wash the column of nonretaining particles before starting a linear NaCl gradient (50 mM to 350 mM).

Fractions containing MDO were pooled based on SDS-PAGE and concentrated to ∼50 ml using an Amicon stir cell and YM-10 ultrafiltration membrane. The C-terminal His-tag was removed by thrombin protease (Bio pharma Laboratories) overnight at 4 °C with gentle stirring. Tag-free protein was then concentrated to ∼1% of the S100 Sephacryl (info on sourcing) size exclusion column volume before being loaded directly onto packing preequilibrated with 20 mM Hepes, 50 mM NaCl, pH 8.0. Fractions were eluted at a speed of ∼0.8 ml/min and pooled based on SDS PAGE analysis as described elsewhere (26). The enzyme solution was then concentrated with Amicon 10 kDa spin concentrators at 5000 rpm to ∼ 5 ml before freezing in liquid nitrogen and storing at −80 °C. Iron content was determined for both ferric and ferrous concentration using 2,4,6-tripyridyl-s-triazine in a method previously described (33).

### Isotopically labeled substrates

Deuterium enriched [99%] 3-mercaptopropionic acid (d_4_-3MPA, 2,2,3,3-^2^H-3MPA) was purchased from CDN Isotopes (p/n D-7909). Alternatively, 3MPA selectively ^2^H-labeled [>95%] at C2 (d_2_-3MPA, 2,2,-^2^H-3MPA) was synthesized by members of the Foss group at The University of Texas at Arlington. Both the synthesis of this substrate and its analysis are presented in Supporting Information, Section I, II, Figs. S1−S4).

### EPR samples

Fe(II)-MDO (0.8 mM) samples were prepared with a 5-fold molar excess of 3MPA (4 mM) and a 10-fold excess of NO (8 mM) in 20 mM Hepes, 50 mM NaCl, pH 8.0 buffer. All stock solutions, buffers, and proteins were degassed by argon/vacuum cycling on a Schlenk line before transferring into the anaerobic chamber (Coy Labs). Stock substrate (3MPA) solutions were prepared at 40 mM in anaerobic 200 mM Hepes and 50 mM NaCl at pH 8.0 buffer. NO stock solutions were prepared by the addition of 5.5 mg PROLI NONOate (Cayman Chemical) to degassed 10 mM NaOH. To initiate the release of NO, the NONOate/NaOH solution was diluted 1:1 with 200 mM Hepes, 50 mM NaCl, and pH 8.0 buffer. Upon completion, the final concentration of NO in solution was nominally 160 mM. Samples of (3MPA/NO)-MDO were prepared by sequential addition of 3MPA before NO. After the anaerobic addition of substrate, the solution was gently mixed and allowed to equilibrate on ice for 10 min before the addition of NO. Similarly, after the addition of NO, samples were mixed again and allowed to sit on ice for another 10 min before transfer to a 3-mm suprasil quartz EPR tube and freezing in liquid nitrogen.

### Computational modeling

Calculations were performed using Orca version 5.0 (https://orcaforum.kofo.mpg.de/app.php/portal) (71). Starting coordinates for geometry optimizations were extrapolated from the crystal structure of *A. vinelandii* MDO:3HPA complex [PDB code: 6XB9] and *P. aeruginosa* [PDB code: 4TLF]. The alpha carbons for each residue were capped with methyl groups before optimizations. Iron-coordinated histidine residues were capped at the beta-carbon and protonated at the δ-position. As described previously, geometry optimization of the (3MPA/NO)-MDO iron-nitrosyl complexes used the B3LYP functional with the Ahlrichs def2-tzvp basis set on iron and directly coordinated atoms and the def2-svp basis set on all other atoms (72, 73, 74). Capped methyl groups were restricted during the geometry optimization. Additionally, the wavefunction for the coupled *S* = 3/2 spin state of the iron-nitrosyl cluster was calculated using a “broken-symmetry approach” to account for the antiferromagnetic coupling between the *S* = 5/2 for iron and *S* = −1 on the nitrosyl ligand (75). All calculations utilized Grimme’s D3 dispersion correction, a conductor-like polarizable continuum model solvent model with ε = 4 to emulate a protein environment, and resolution of identity and chain of sphere (RIJCOSX) approximation for B3LYP with def2/J auxiliary basis sets (76, 77, 78, 79).

### ENDOR spectroscopy

ENDOR measurements were collected at 5 K using an ELEXSYS E680 EPR spectrometer (Bruker-Biospin) equipped with a Bruker Flexline ER 4118 CF cryostat and an ER 4118X-MD4-ENDOR resonator. Measurements were performed using a microwave frequency of 9.76 GHz at various field positions spanning the low-field edge of the CW EPR spectrum, where ^1^H near the iron center is readily resolved (39). Measurements used the Mims ENDOR pulse sequence, π/2− τ− π/2 − T− π/2−τ−echo, with a 25 μs rf π pulse applied during the delay time T. The delay time τ was set to 120 ns, a value that not only provided the best resolution for measuring proton ENDOR, but also minimized interference from Mims “blind spots”. ENDOR spectra were processed using xEPR (Bruker-Biospin) and simulated using the Easyspin toolbox in MATLAB (80). Simulations took into account the distance and orientation of the protons on 3MPA, Tyr159-OH, and the coordinated histidine residues. In brief, hyperfine tensors for each proton were constructed assuming an axial hyperfine interaction with principal values defined by Equation 1,

(1) $$\left(A_{iso}-T,A_{iso}-T,A_{iso}+2T\right)$$

where $A_{iso}$ and T are the isotropic and anisotropic (dipolar) contributions to the hyperfine coupling, respectively. In similar iron-nitrosyl sites, the isotropic component of ^1^H hyperfine couplings is assumed to be either small (<2 MHz) or zero because of the small or absent Fermi contact interaction between the unpaired spin and protons in the first and second coordination spheres (51, 52). For all simulations, $A_{iso}$ was assumed to be zero, as the dipolar coupling largely determined the peak position in simulated spectra. Since the spin in a iron-nitrosyl cluster is delocalized across the Fe, N, and O atoms, the overall dipolar coupling of a given proton is a function of the individual dipolar couplings between the proton and each atom within the Fe-NO bond. These couplings can be calculated using the dipole-dipole model (Equation 2), which considers the distances between the proton and each atom,

(2) $$T_{Fe,N,or\;O}=\left(\frac{\mu_{0}}{4\pi }\right)\frac{g_{e}g_{n\beta_{e}\beta_{n}}}{hr^{3}}$$

where $\mu_{0}$ is the permittivity of free space, $g_{e}$ is the electronic *g-value*, $g_{n}$ is the nuclear *g-value*, $\beta_{e}$ is the electronic Bohr magneton, $\beta_{n}$ is the nuclear Bohr magneton, h is Planck’s constant, and r is the distance in nm from the proton to the Fe, N, or O. Distances between each proton and the Fe, N, and O in the Fe-NO bond were measured using the geometry-optimized model for the (3MPA/NO)-MDO ternary complex. The overall dipolar coupling of a given proton to the spin center can then be calculated with spin projection factors (Equation 3), as has previously been described (51, 81) The overall dipolar coupling, T, is calculated as

(3) $$T=\frac{7}{5}\;\left(T_{Fe}\right)-\;\frac{1}{5}\left(T_{N}+T_{O}\right)$$

where $T_{Fe}$, $T_{N}$, and $T_{O}$ are the dipolar couplings between the proton and the Fe, N, and O, respectively. In our simulations, the calculated overall dipolar couplings for each proton were varied within the resolution of the crystal structure (0.1 Å) that the geometry-optimized model was based on. The optimal *T* value for each proton was chosen based on how well the simulated peaks for each matched the experimentally observed peaks. The calculated *T* and the optimal *T* for each proton are listed in Supporting Information, Section III, Table S1.

In addition to the overall dipolar coupling, the orientations of each proton were simulated using Euler rotations that related the ^1^H-Fe vector to the magnetic coordinate system. Due to the near-axial symmetry of the EPR spectrum and the fact that the principle axis of the zero-field splitting is defined by the Fe-NO bond, only two angles are needed to relate the ^1^H-Fe vector to the magnetic coordinate system (51). Consequently, Euler rotations can be approximated using polar angles φ and θ, where φ is taken as the angle between the proton and the xz-plane, and θ defines the deviation from the z-axis defined by the Fe-NO bond. Fig. S5 shows how θ and φ are related to the magnetic axis system. Values for θ and φ were measured from the geometry-optimized structure using PyMol (version 2.3.3, https://pymol.org/2/). Simulations were largely insensitive to the value of φ and dominated by the value of θ. For protons on C2 of 3MPA, θ was varied up to 10% to obtain the best match between experimental and simulated peaks. Values for θ for the protons on C3 of 3MPA were used directly as measured. All Euler rotation parameters can be found in Supporting Information, Section III, Table S2. Processed ENDOR data and simulations were plotted using Spincount (version 6.2, http://www.chem.cmu.edu/groups/hendrich/facilities/index.html), developed by Professor Michael Hendrich at Carnegie Mellon University. Simulation intensities were scaled by a uniform scalar to match the peak intensity of difference spectra across all measured magnetic fields.

## Data availability

The atomic coordinates for the *cis*-C3 (3MPA/NO)-MDO computational model are provided in supporting information. All other molecular coordinates, computational results, and raw spectroscopic data used in this study will be made available upon request (Contact: Brad Pierce, bspierce1@ua.edu).

## Supporting information

This article contains supporting information (26, 33, 39, 82, 83, 84).

## Conflict of interest

The authors declare that they have no conflicts of interest with the contents of this article.

## Supporting information

Supporting Information Sections I-XII, Supplemental Figures S1–S15 and Tables S1–S3
