# Supplementary material for: Improved resolution of 3-mercaptopropionate dioxygenase active site provided by ENDOR spectroscopy offers insight into catalytic mechanism
Source: J Biol Chem. 2024 Feb 21;300(4):105777. doi: 10.1016/j.jbc.2024.105777 (PMC10966181; doi:10.1016/j.jbc.2024.105777)
Supplement: Supporting Information Sections I-XII, Supplemental Figures S1–S15 and Tables S1–S3 [file mmc1.pdf]

Supporting Information

Improved resolution of 3-mercaptopropionate dioxygenase active site provided by ENDOR spectroscopy offers insight into catalytic mechanism

Brad S. Pierce[1], Allison N. Schmittou[1], Nicholas J. York[1], Ryan P. Madigan[3], Paula F. Nino[3], Frank W. Foss, Jr[3], Molly M. Lockart[2*]*

1 Department of Chemistry & Biochemistry, University of Alabama, 250 Hackberry Lane, Tuscaloosa, Alabama 35487, United States

2 Department of Chemistry and Biochemistry, Samford University, 800 Lakeshore Drive, Homewood, AL 35229, United States

3 Department of Chemistry & Biochemistry, The University of Texas at Arlington, 700 Planetarium Place, Arlington, TX 76019 United States

Table of Contents for Supplementary Information

Section	Description	Page
**I**	Synthesis of deuterated derivatives	2-3
**II**	^{1}H and ^{13}C{^{1}H} NMR Spectra	4-6
**III**	ENDOR simulation parameters	7-8
**IV**	van der Waal model of (**3MPA**/NO)-MDO	9
**V**	Additional ENDOR difference spectra obtained with d_2-**3MPA**	10
**VI**	Additional ENDOR difference spectra obtained with d_4-**3MPA**	11
**VII**	Comparative HYSCORE simulations for *cis*-C3 and *trans*-C3 **3MPA**-conformations	12-13
**VIII**	Representative CW EPR spectra of WT and H157N (**3MPA**/NO)-MDO	14
	Additional ENDOR difference spectra and simulations of WT versus H157N (**3MPA**/NO)-MDO	15
**IX**	ENDOR simulations of coordinated histidine rotations	16
**X**	Temperature-dependent kinetic experiments	17
**XI**	Optimized models for the **3MPA**-bound enzyme	18
**XII**	References	23

I. Synthesis of Deuterated Derivatives

General Comments. All reagents and solvents were purchased from Sigma Aldrich, VWR, or Fisher Scientific. All reactions were carried out under a nitrogen atmosphere with anhydrous solvents. ^{1}H NMR spectra were acquired on 500 MHz JEOL spectrometers and referenced to the internal solvent signals (7.26 ppm in $CDCl_3$ or 3.31 ppm in CD_3OD). ^{13}C{1H} NMR spectra were acquired on a 125 MHz spectrometer referenced to the internal solvent signals with a central peak at 77.00 ppm in $CDCl_3$, except for the final product which was acquired on a 100 MHZ spectrometer referenced to CD_3OD at 49.00 ppm. NMR data are reported as follows: chemical shift (in ppm, δ), integration, multiplicity (s = singlet, d = doublet, t = triplet, q = quartet, m = multiplet, br = broad), coupling constant (in Hz, *J*). Thin layer chromatography was performed on silica gel-coated aluminum plates (EMD Merck F254, 250μm thickness). Ultraviolet lights (254 nm) were used to visualize components. Additional stains were used to develop plates where needed, including ninhydrin and phosphomolybdic acid staining agents. Flash column chromatography was performed over Silicycle SilicaflashP60 silica gel (normal phase, mesh 230-400) or Silicycle SiliaBond C18 (reverse phase, mesh 40-63). IR spectra were recorded in a Bruker Alpha-P FT-IR Spectrometer by attenuated total reflectance on a diamond sample plate. HRMS data were recorded by LCMS-IT-TOF in the Shimadzu Center for Advanced Analytical Chemistry at UT Arlington.

Figure S1.

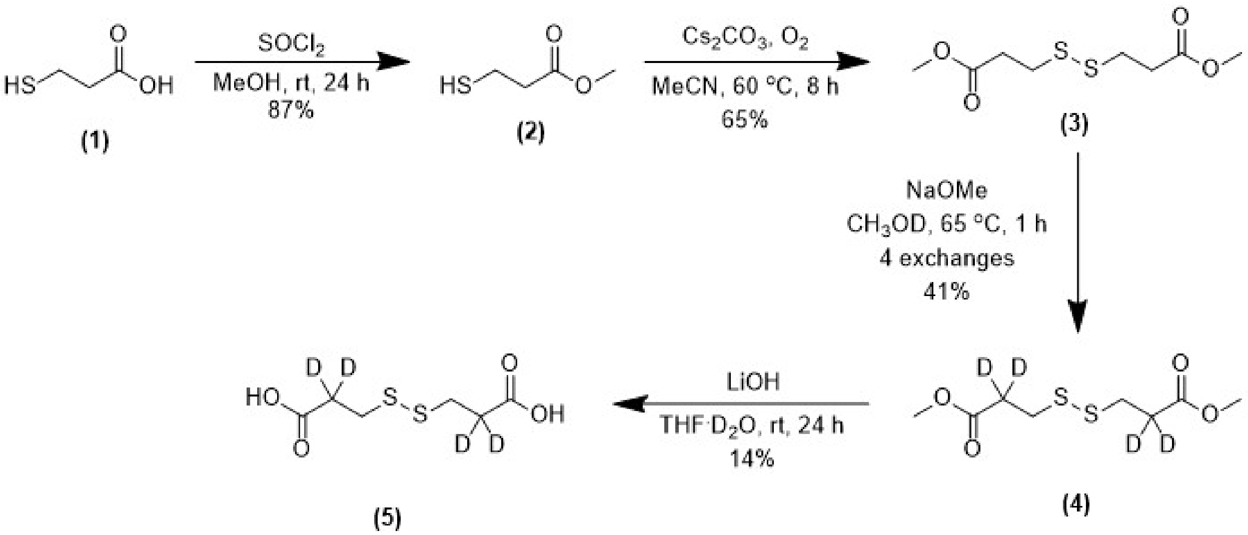

Figure S1. Synthetic route for 3 3'-dithio-4 4’ 4D-dipropionic acid

Synthesis of methyl 3-mercaptopropionate **(2)**[1-3]

3-mercaptopropionic acid (**1**) (2.472 g, 0.023 mol) was added to methanol (9.5 mL) on an ice-water bath. Thionyl chloride (0.1 mL, 0.5 mmol) was added to the reaction dropwise while stirring. The reaction was allowed to come to room temperature while stirring over 24 hours. The methanol solution was evaporated and chloroform was added to the crude mixture, which was then washed with a NaOH solution adjusted to pH 9-10, brine, and

then dried over Mg_2SO_4, before being concentrated giving the product methyl (3-mercapto)propionate as a clear oil (1.425 g, 51%). ^{1}H NMR (500 MHz, $CDCl_3$) δ 6.37-6.29 (m, 2H), 6.22 (t, *J*=6.7 Hz, 2H), 5.23 (t, *J*=8.3 Hz, 1H); ^{13}C{^{1}H} NMR (125 MHz, $CDCl_3$) δ; 171.6, 51.3, 37.9, 19.3 IR (neat, cm^{-1}): 2570.51, 1731.27, 1200.71; HRMS (ESI) calcd for $C_4H_8O_2S$ $[M+H]^+$; predicted: 121.0245, found: 121.0295.

Synthesis of Dimethyl 3,3′-dithiobispropanoate **(3)**
Compound **2** (1.425 g, 0.012 mol) was added to a solution of acetonitrile (29 mL) and cesium carbonate (0.73 mg, 0.24 mol) and refluxed for 8 hours while being open to air. Following the reaction time, acetonitrile was removed under reduced pressure and DI water was added and subsequently extracted with ethyl acetate three times. The combined organic layers were dried with sodium sulfate and concentrated. Purification was carried out by column chromatography (0-15% Ethyl Acetate:Hexane) to give a clear oil (0.928 g, 65%). ^{1}H NMR (500 MHz, $CDCl_3$) δ; 3.53 (s, 3H), 2.64 (t, *J*=7.3 Hz, 2H), 2.46 (t, *J*=7.4 Hz, 2H); ^{13}C{^{1}H} NMR (125 MHz, $CDCl_3$) δ; 171.7, 51.2, 34.2, 26.5 IR (neat, cm-1): 2846.52, 1729.96, 1168.92; HRMS (ESI) calcd for $C_8H_{14}O_4S_2$ $[M+H]^+$; predicted: 239.0334, found: 239.0359.

Deuteration of Dimethyl 3,3′-dithiobispropanoate **(4)**
Disulfide **3** (0.473 g, 2 mmol) was added to CH_3OD (4.5 mL, 0.1 mol) and freshly prepared sodium methoxide was made by dissolving solid sodium in methanol (0.4679 g, 2.4 mmol) and refluxed for one hour at 65 °C. Following reflux, methanol was evaporated off and fresh CH_3OD was added and the solution was refluxed again for an additional hour. This process was repeated four times, with the last time being quenched with deuterated trifluoracetic acid until the pH equaled 1. The solution was evaporated until all methanol and TFA was removed, reconstituted in DI water, and washed three times with dichloromethane. The organic layers were combined and then dried with magnesium sulfate before concentration under reduced pressure. The crude mixture was further purified by column chromatography (0-15% Ethyl Acetate:Hexane) to receive an impure yellow oil (0.198 g). The crude product containing compound **4** was used without further purification in the next reaction.

Synthesis of 3 3'-dithiodipropionic acid **(5)**
Crude mixture **4** (0.256 g) was added to THF (6.2 mL) over an ice-water bath. A solution of LiOH (0.098 g) in D_2O (4.25 mL) was added to the above solution and allowed to stir for 30 minutes, before being allowed to warm up in which it was stirred for an additional for 24 hours. The reaction was acidified using D-TFA, and then excess THF and D-TFA were removed in vacuo. The remaining aqueous solution was basified to a pH of 9-10 with NaOH and washed three times with ethyl acetate, with the organic layers being discarded. The aqueous solution was then acidified with HCl to a pH = 1 and extracted with ethyl acetate three times. The organic layers were dried with sodium sulfate, and then purified through a reverse phase column (2:8 ACN:Water). The product was produced as a white crystalline solid (0.156 g). ^{1}H NMR (500 MHz, CD_3OD) 2.91 (s, 4H); ^{13}C{^{1}H} NMR (100 MHz, CD_3OD) δ; 175.49, 34.11, 27.68 IR (neat, cm-1) 3072, 2980, 2927, 2809, 2678, 2575, 2127, 1687, 1406, 1290, 1243, 1209, 1132, 1036, 968; HRMS (ESI) calcd for $C_6H_6D_4O_4S_2$ [M-H]-; predicted: 213.0272 found: 213.0199

II. **^{1}H and ^{13}C{^{1}H} NMR Spectra**

Figure S2. NMR spectroscopic characterization of synthetic product (**2**)

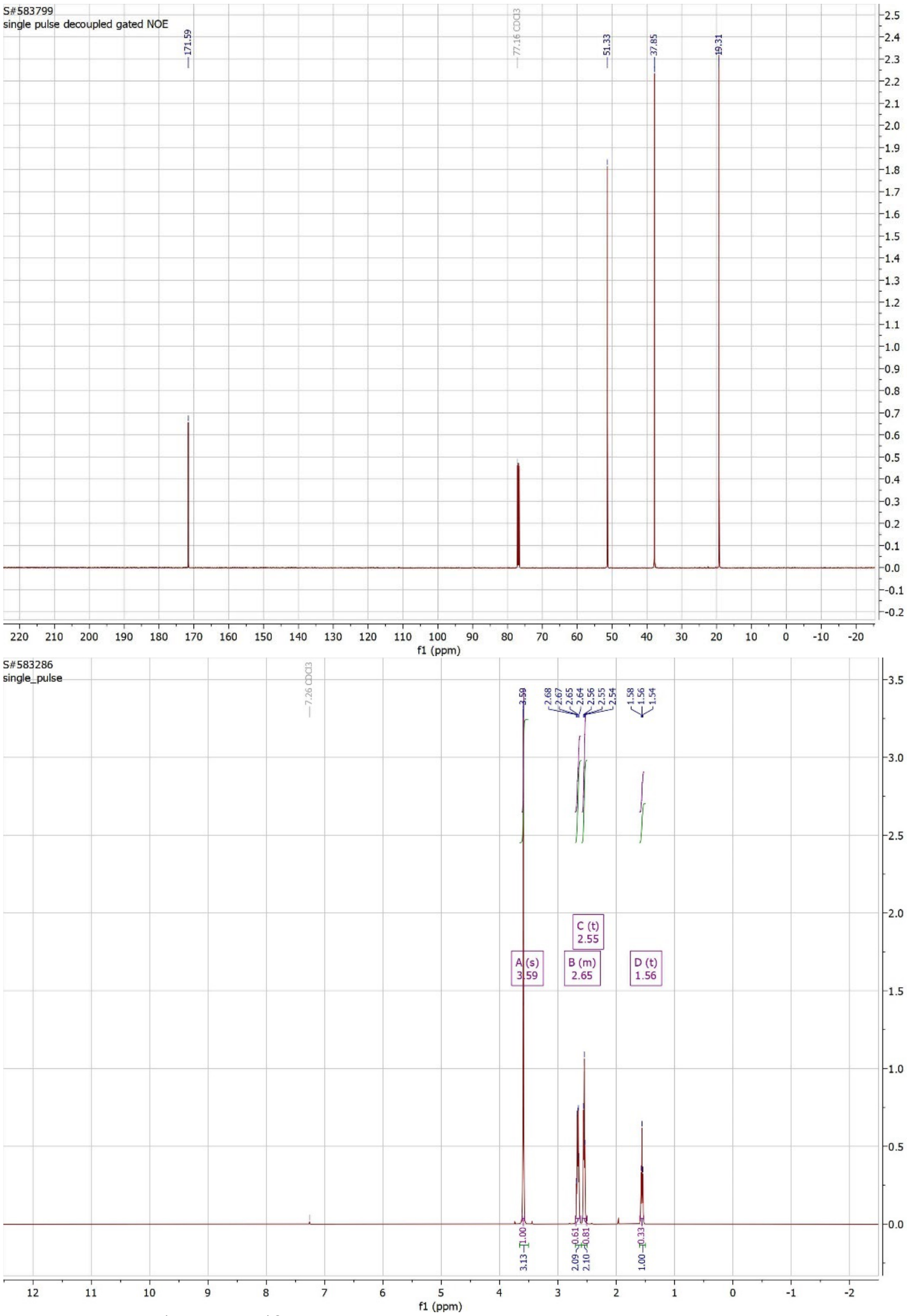

Figure S2. ^{1}H and ^{13}C spectral figure of **2**

Figure S3. NMR spectroscopic characterization of synthetic product (**3**)

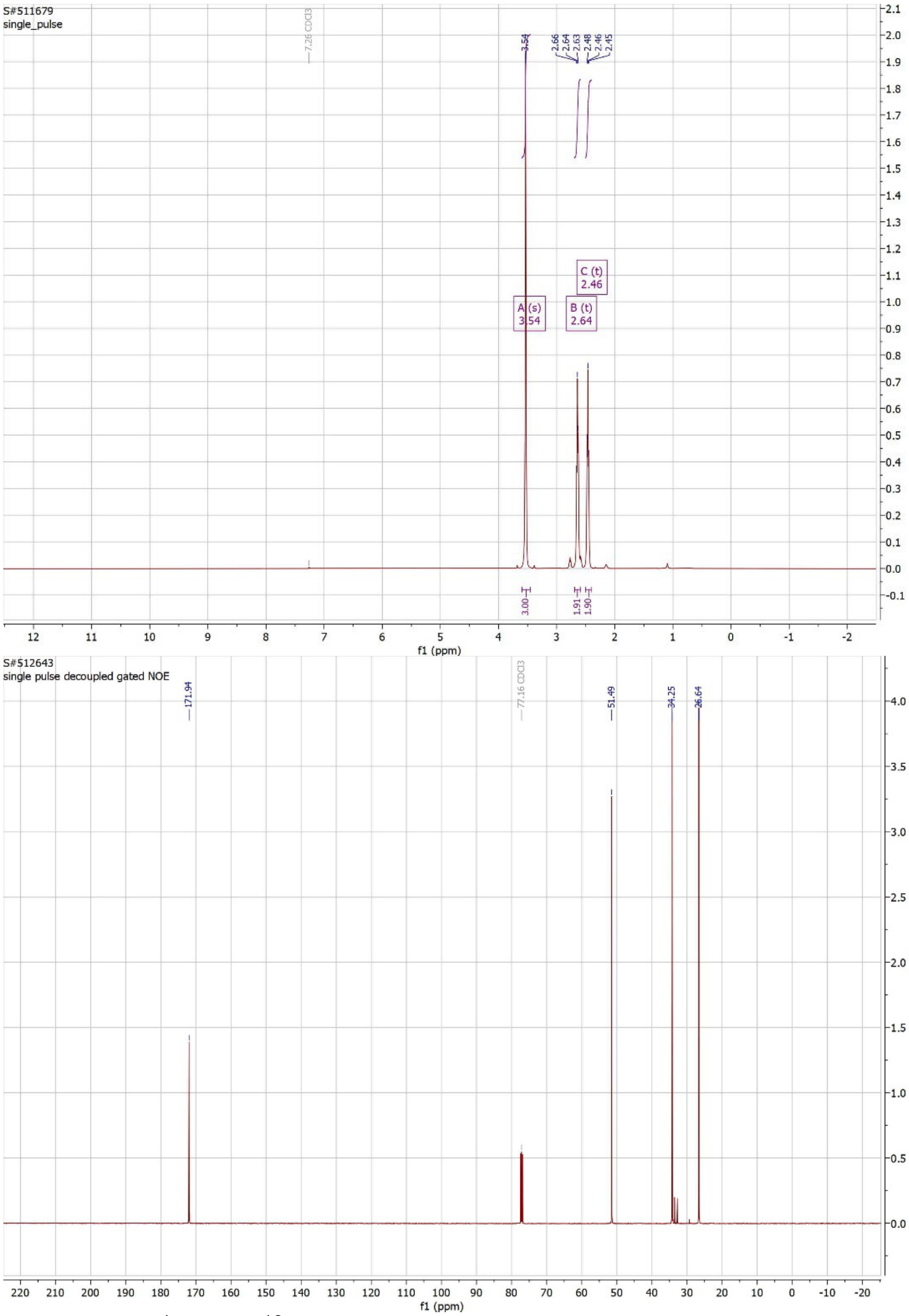

Figure S3. ^{1}H and ^{13}C spectral figure of **3**

Figure S4. NMR spectroscopic characterization of synthetic product (**5**)

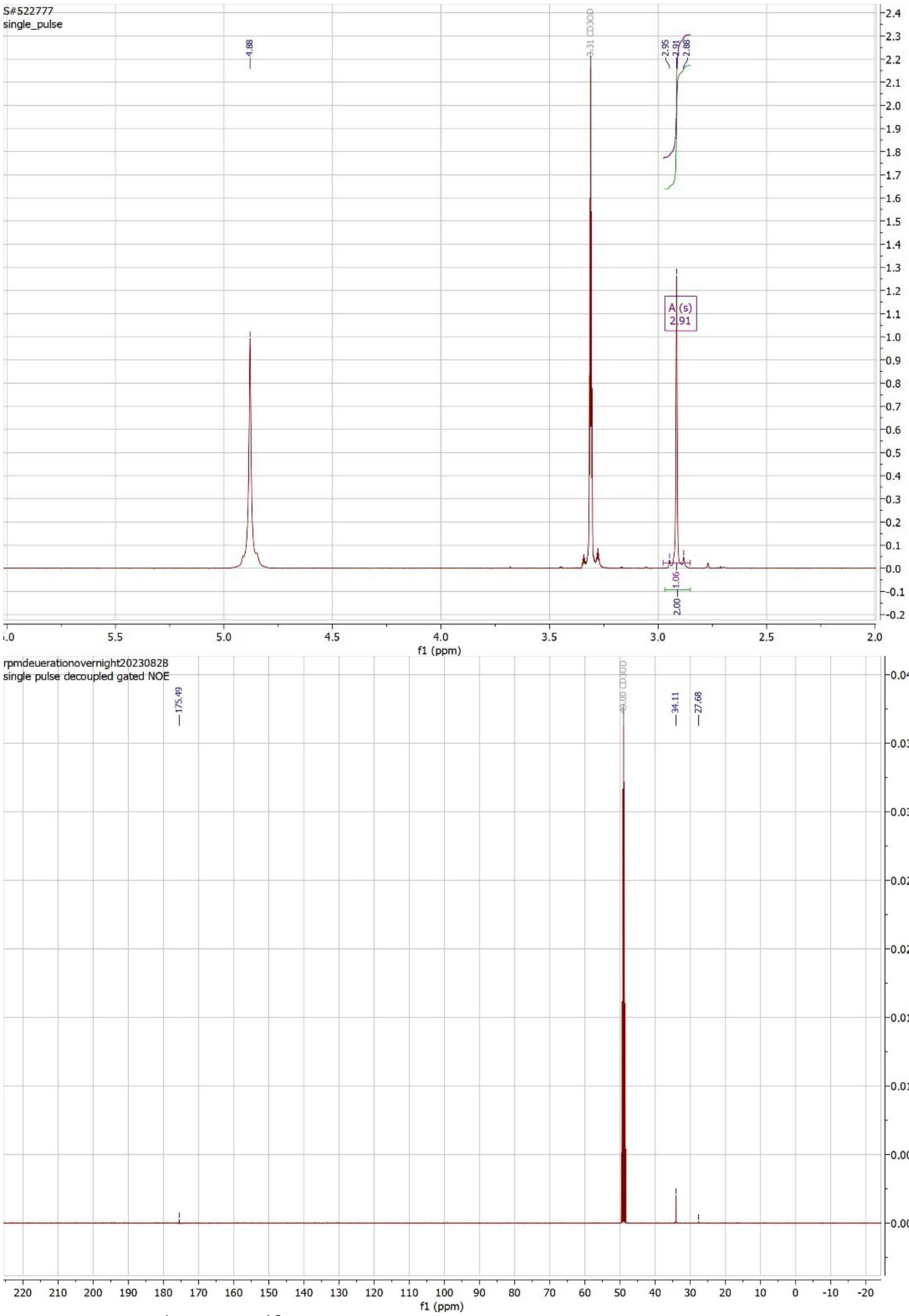

Figure S4. ^{1}H and ^{13}C spectral figure of **5**

III. ENDOR Simulation Parameters

Figure S5. The magnetic coordinate system for the *cis*-C3 (**3MPA**/NO)-MDO illustrates the relative placement of selected protons to the iron-nitrosyl.

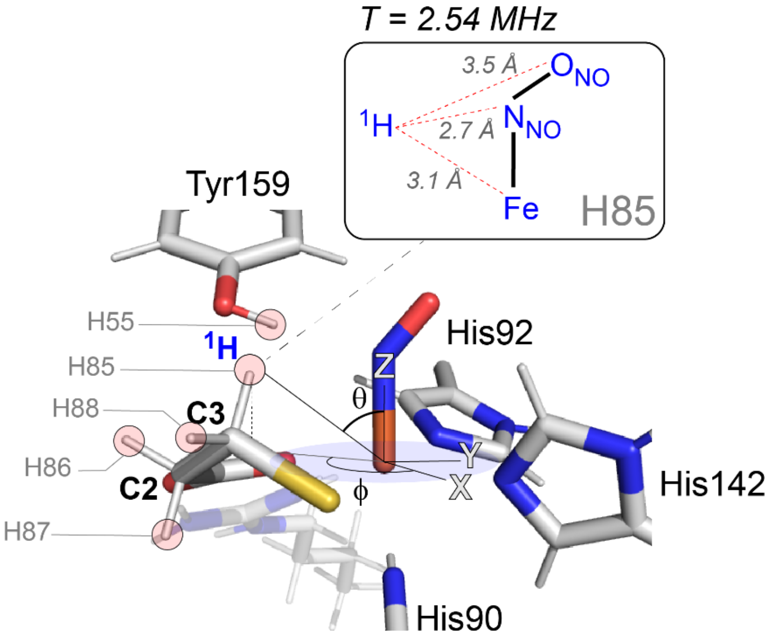

Figure S5. Model of the (**3MPA**/NO)-MDO active site showing how the angles θ and φ relate the position of a proton to the calculated magnetic axis system. The box highlights how the distances between a proton and the Fe, N, and O of the Fe-NO center are measured. Using these distances, dipolar couplings for the ^{1}H-Fe, ^{1}H-N, and ^{1}H-O can be calculated using the dipole-dipole approximation (**Eq**. **2**). These individual dipolar couplings are then used to calculate an overall dipolar coupling, *T*, for each proton using (**Eq**. **3**). The proton labels correspond to their atom numbers in the overall geometry-optimized structure. Protons H85-H88 are on **3MPA**, and H55 is on the Y159-OH. An analogous coordinate system model was generated for (*trans*-C3, conformer ***2***) to related changes in C3 proton placement.

Table S1. Distances and dipolar couplings for all protons included in the ENDOR simulations. Difference spectra simulations of d$_2$-**3MPA** included the protons on the C2 of **3MPA** (H85 and H88). Difference spectra simulations of d$_4$-**3MPA** included all substrate protons (H85-H88), and simulations of the H157N difference spectra included the proton on Y159-OH (H55).

	Distance to Fe-NO atoms			*Intrinsic couplings*				
Proton	*^{1}H-Fe (Å)*	*^{1}H-N (Å)*	*^{1}H-O (Å)*	*T_{Fe} (MHz)*	*T_N (MHz)*	*T_O (MHz)*	*Overall T* (MHz)	*Optimal T (MHz)*
H85	3.60	3.70	4.70	1.69	1.56	0.76	1.91	1.75
H86	4.30	5.10	6.30	0.99	0.60	0.32	1.21	1.17
H87	3.50	4.90	6.00	1.84	0.67	0.37	2.37	2.25
H88	4.60	5.20	6.30	0.81	0.56	0.32	0.96	0.90
H55	3.10	2.00	2.70	2.65	9.88	4.02	0.94	0.94

Table S2. Euler rotation angles for all protons included in the ENDOR simulations.

Proton	T (MHz)	θ (°)	ϕ (°)
H85	1.75	84	-100
H86	1.17	106	-85
H87	2.25	127	-85
H88	0.90	104	-100
H55	0.94	39	-20

Table S3. Comparison of dipolar couplings (*T*) and Euler rotation angles for protons of **3MPA** between *cis*-C3 and *trans*-C3 conformers.

Proton	T (MHz)	θ (°)	ϕ (°)
cis-C3 H85	0.98	100	-115
trans-C3 H85	1.75	84	-100
cis-C3 H86	1.16	79	-115
trans-C3 H86	1.17	106	-85
cis-C3 H87	2.54	59	-120
trans-C3 H87	2.25	127	-85
cis-C3 H88	1.52	110	-115
trans-C3 H88	0.90	104	-100

IV. van der Waal model of (3MPA/NO)-MDO

Figure S6. Space fill model of computational model ***2***.

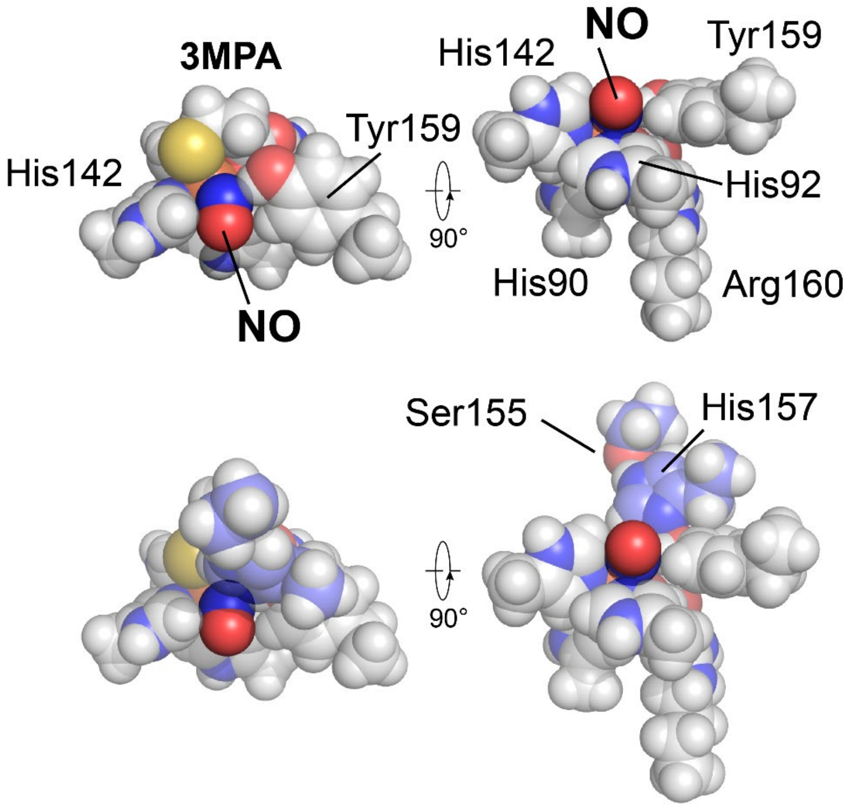

Figure S6. Optimized model of the *trans*-C3 (**3MPA**/NO)-bound MDO site illustrating the NO (*and dioxygen*) binding pocket. On top only Tyr159 of the SHY motif is shown for clarity. Below, His157 and Ser155 are included (*in blue*) to illustrate how these residues further restrict dioxygen binding geometry at the Fe-site. The sphere scale for all atoms is set to the default atomic radii for each atom. In the above figure, H, N, O, and S-atoms are colored white, blue, red, and yellow, respectively.

V. Additional ENDOR difference spectra obtained with d_2-3MPA

Figure S7. Comparison of simulations overlaid on field-dependent ENDOR difference spectra (natural isotopic abundance versus d_2-**3MPA**).

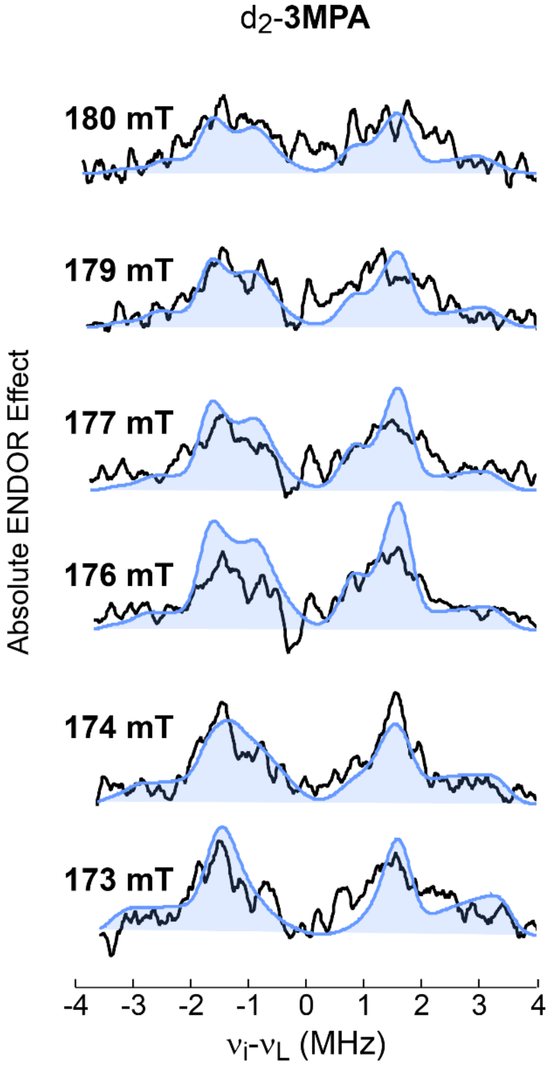

Figure S7. [^{1}H-^{2}H] ENDOR difference spectra obtained for (**3MPA**/NO)-bound MDO at six field positions (*black traces*). Difference spectra were obtained by subtracting ENDOR spectra obtained with d_2-**3MPA** from samples prepared with natural isotopic abundance. Field-dependent simulations for **3MPA** C2-protons (*blue trace*) are overlaid on difference spectra for comparison. Distance and orientations of C2 protons were taken from DFT-optimized models. Since the position of C2 protons is not significantly altered in *cis*-C3 and *trans*-C3 isomerization, simulations generated from either optimized conformer (***1*** or ***2***) fit equally well.

VI. Additional ENDOR difference spectra obtained with d_4-3MPA

Figure S8. Comparison of simulations generated from conformer **1** and **2** models overlaid on field-dependent ENDOR difference spectra (natural isotopic abundance versus d_4-**3MPA**).

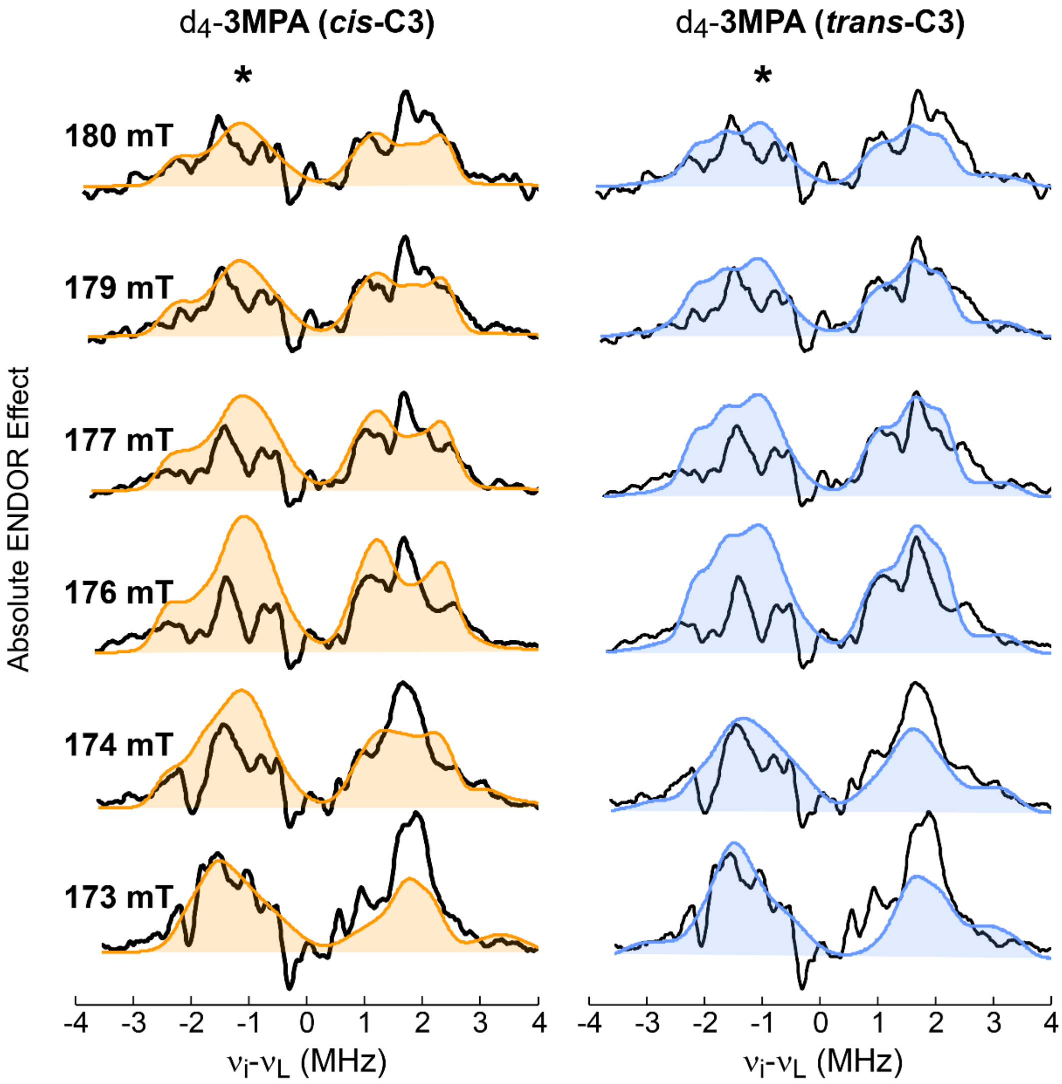

Figure S8. [^{1}H-^{2}H] ENDOR difference spectra obtained for (**3MPA**/NO)-bound MDO at six field positions (*black traces*). Difference spectra were obtained by subtracting ENDOR spectra obtained with d_4-**3MPA** from samples prepared with natural isotopic abundance. Field-dependent simulations for **3MPA** C2 and C3-protons are overlaid on difference spectra for comparison. Distance and orientations of C2 and C3 protons were taken from DFT-optimized models (***1***, *cis*-C3, orange) and (**2**, *trans*-C3, blue). The region of the spectra suppressed by implicit TRIPLE effect marked (*) for clarity.

VII. Comparative HYSCORE simulations for *cis*-C3 (*1*) and *trans*-C3 (*2*) 3MPA

Figure S9. Field-dependent ^{1}H HYSCORE simulations generated from computational model ***1***.

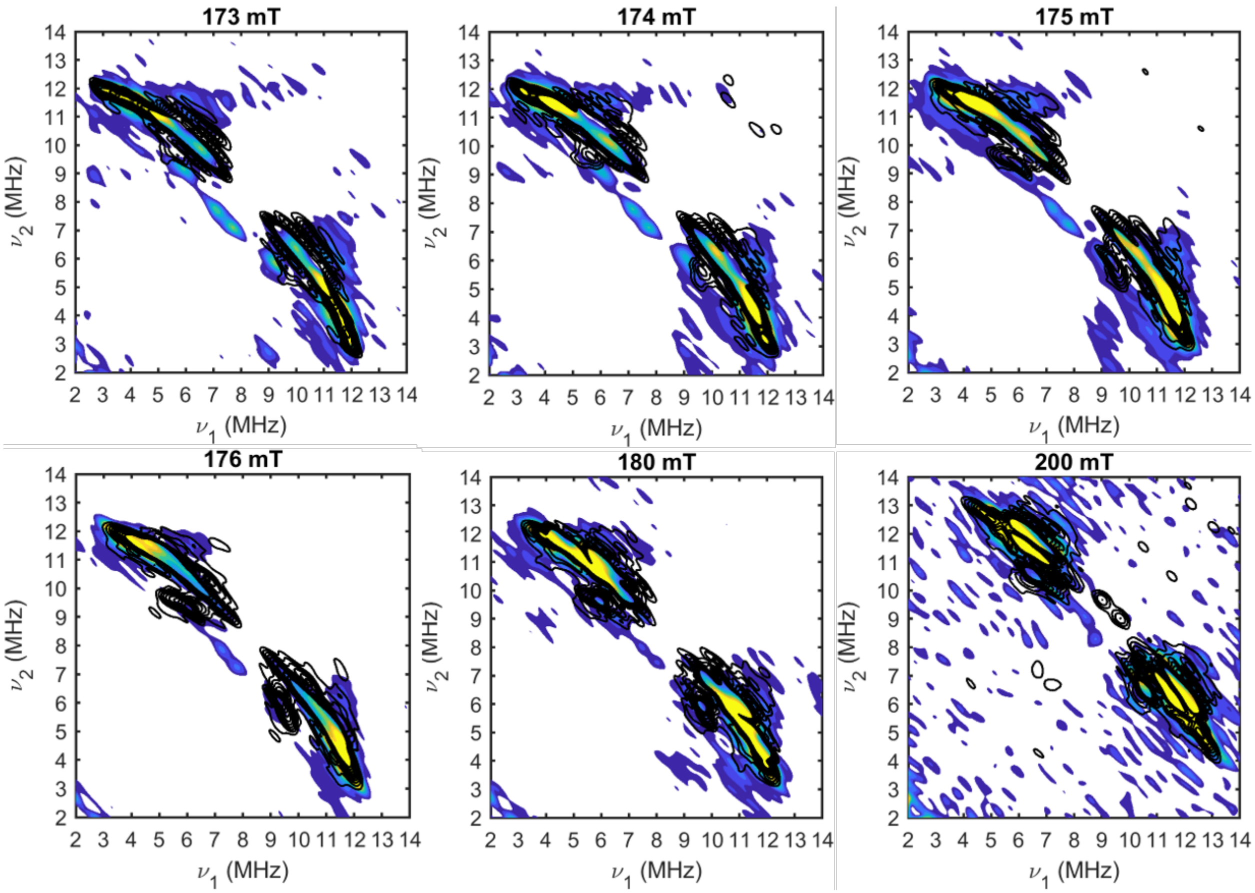

Figure S9. Comparison of (**3MPA**/NO)-MDO HYSCORE data (color contours) collected at six field positions (173 – 200 mT) overlaid with simulations (black contours) that used parameters from the DFT optimized mode for (*cis*-C3-**3MPA**/NO)-MDO. This figure is presented in our previous work.[4]

Figure S10. Field-dependent ^{1}H HYSCORE simulations generated from computational model ***2***.

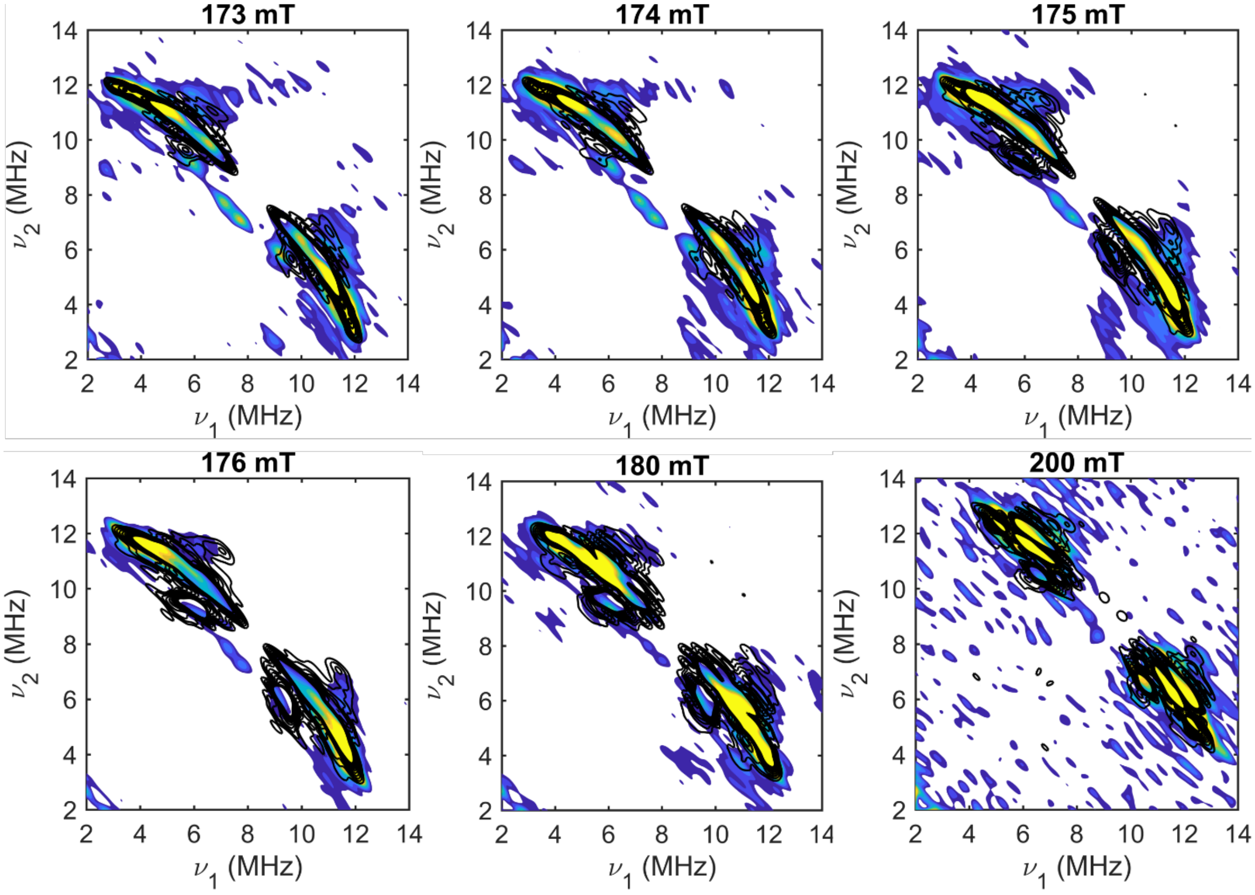

Figure S10. Overlay of the previously presented experimental HYSCORE data[4] (color contours) with new simulations (black contours) using parameters from the DFT optimized model for (*trans*-C3-**3MPA**/NO)-MDO.

VIII. Representative CW EPR spectra of WT and H157N (3MPA/NO)-MDO

Figure S11. Representative CW EPR spectra for wild-type and H157N (**3MPA**/NO)-bound enzyme.

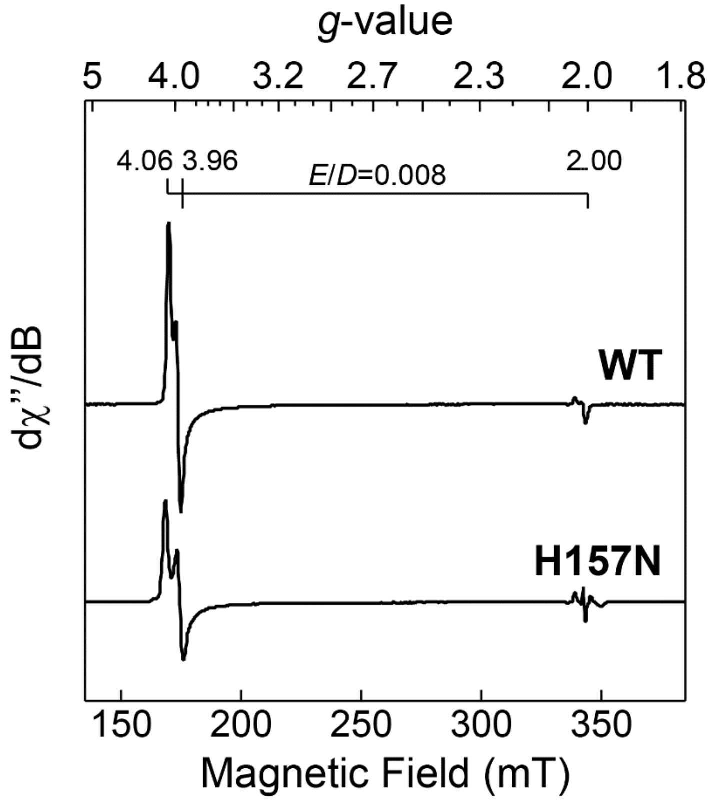

Figure S11. X-Band CW EPR spectra of iron-nitrosyl species produced upon addition of NO to the **3MPA**-bound wild-type (*top*) and H157N MDO variant (*bottom*). Instrumental parameters: microwave frequency, 9.643 GHz; microwave power, 20 μW; modulation amplitude, 0.9 mT; temperature, 10 K.

Additional ENDOR difference spectra and simulations of WT versus H157N (3MPA/NO)-MDO

Figure S12. Simulation of Tyr159 phenol proton overlaid on field-dependent ENDOR difference spectra (wild-type versus H157N MDO variant).

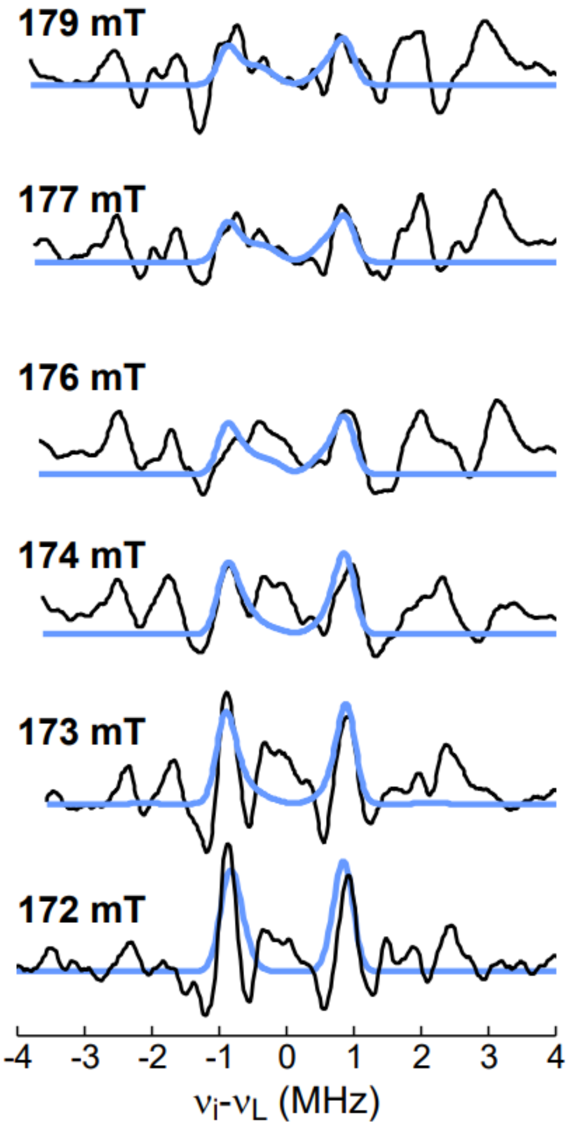

Figure S12. ^{1}H ENDOR difference spectra obtained at selected fields by subtraction of H157N from wild-type (**3MPA**/NO)-bound MDO (*black trace*). For comparison, simulations for the Tyr159 phenol proton (*blue*) are overlaid on difference spectra. Simulations include the distances and orientations of the Try159-OH proton taken from the *trans*-C3 optimized model.

IX. ENDOR simulations of coordinated histidine rotations

Figure S13. Simulation of His142 rotation overlaid on field-dependent ENDOR difference spectra (wild-type versus H157N MDO variant).

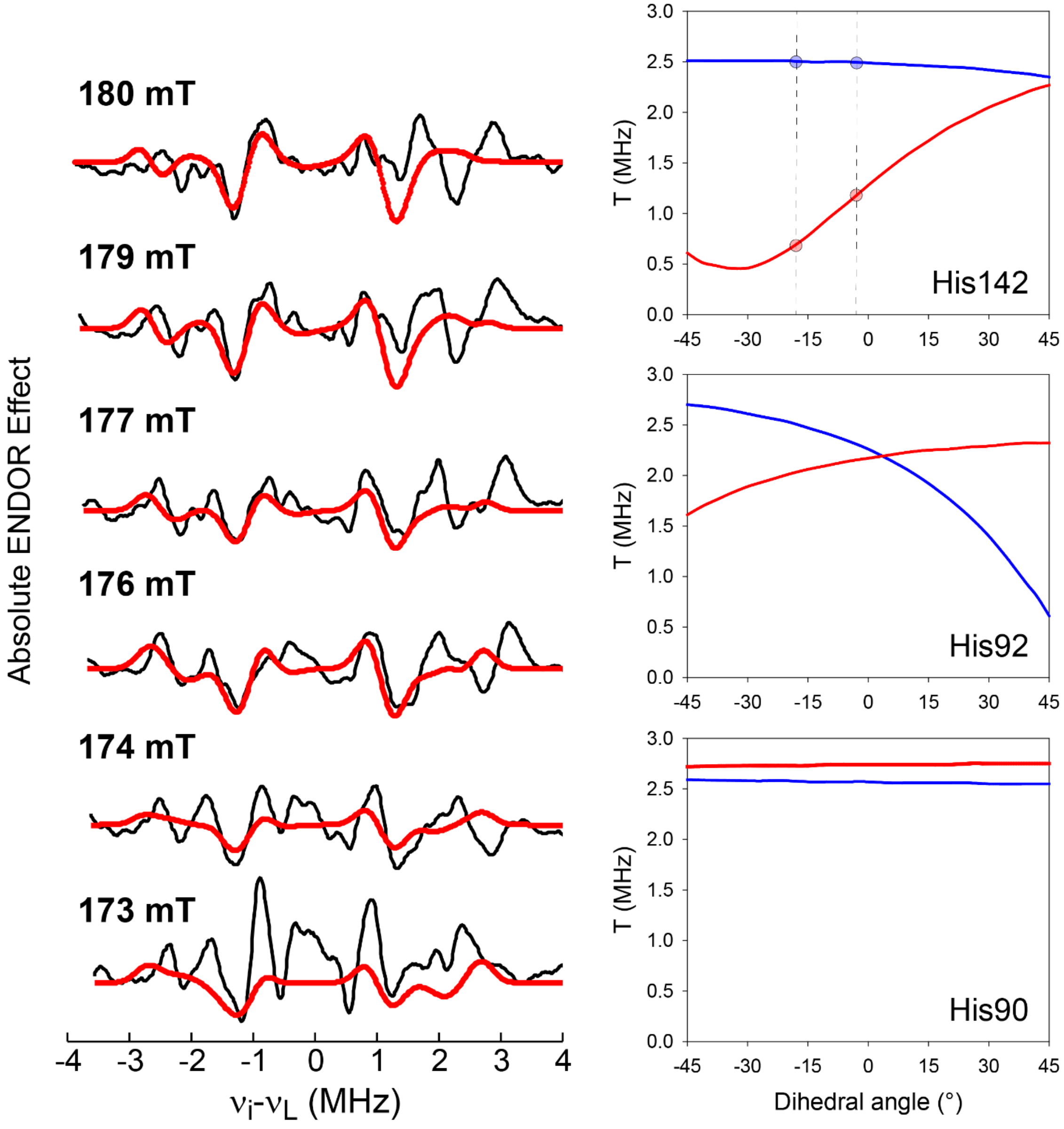

Figure S13. ^{1}H ENDOR difference spectra (*left*) obtained at selected fields by subtraction of H157N from wild-type (**3MPA**/NO)-bound MDO (*black trace*). Here, simulations were calculated assuming the H157N variant exhibits a -15-degree rotation of His142 relative to the wild-type enzyme (*red*). The change in dipolar couplings for His142, His92, and His90 protons (*right*) was calculated based on distances obtained from the trans-C3 optimized model.

X. Temperature-dependent kinetic experiments

The rate of dioxygen consumption was determined polargraphically using a standard Clark electrode (Hansatech Instruments, Norfolk, England) in a jacketed 2.5 mL cell. The electrode was bathed in a saturated solution of KCl and separated from the buffer using a gas-permeable membrane. Before use the electrode was calibrated as recommended by the product manufacturer.

Reaction temperatures ranging from 5-45 ± 2 °C were maintained via a circulating water bath (Grant Instruments). Wild-type or H157N MDO (5.0 μM) was allowed to incubate in the electrode chamber for 60 seconds before reaction initiation by addition of substrate. Temperature-dependent kinetic data were interpreted by transition state theory (TST) and fit to the Eyring equation (Eq. S1) to obtain the enthalpy ($\Delta H^{\ddagger}$) and entropy ($\Delta S^{\ddagger}$) of activation.

$$\ln\frac{k}{T} = \frac{-\Delta H^{\ddagger}}{R}\frac{1}{T} + ln\frac{k_B}{h} + \frac{\Delta S^{\ddagger}}{R} \qquad \textbf{Equation S1}.$$

In this equation, T is the temperature in Kelvin, R is the gas constant 8.314 J·K^{-1}mol^{-1}, h is Planck's constant, k_B is Boltzmann's constant, and *k* is the observed rate constant (k_{cat}).

Figure S14. Eyring plot for MDO and H157N MDO variant.

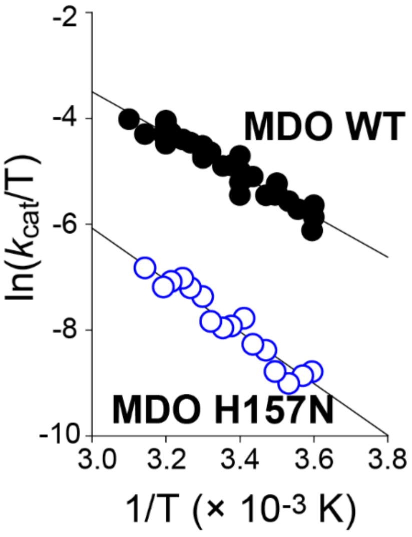

Figure S14. Eyring plot for wild-type (*black, closed circle*) and H157N MDO variant (*blue, open circle*). A summary of experimentally obtained pKa-values[5], $\Delta H^{\ddagger}$, and $\Delta S^{\ddagger}$ for wild-type and H157N MDO variant are presented below.

**Enzyme:substrate**	**pKa$_1$**	**pKa$_2$**	**ΔH‡** (kJ·mol^{-1})	**ΔS‡** (J·mol^{-1}·K^{-1})
CDO:**CYS**[6]	7.6 ± 0.1	10.2 ± 0.1	39 ± 3	-117 ± 8
MDO:**3MPA**	7.8 ± 0.1	9.2 ± 0.1	34 ± 2	-125 ± 9
H157N MDO:**3MPA**	-	9.9 ± 0.1	41 ± 2	-126 ± 2

XI. Optimized models for the 3MPA-bound enzyme

Figure S15. *Optimized models for **3MPA**-MDO with axial chloride, hydroxide, and water.*

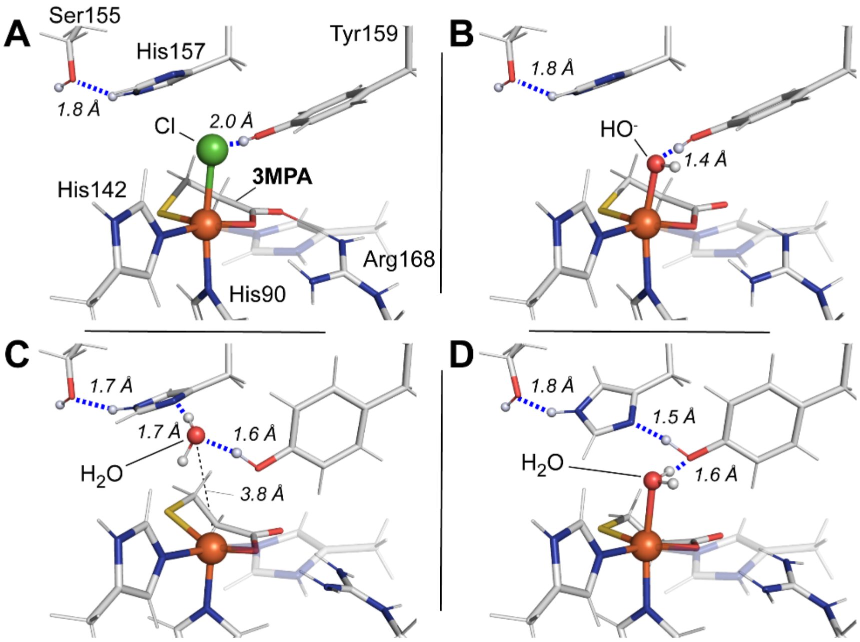

Figure S15. Structures for optimized DFT models illustrating the most stable configuration for the axial Fe-bound chlorine (**A**), and hydroxide (**B**) structures. For both **A** and **B**, the most stable configuration occurs with Tyr159 donating an H-bond to the axial ligand. By contrast, Tyr159 H-bond donation to the axial Fe-bound aqua ligand (**C**) promotes the dissociation of water. Only by switching the Tyr159 H-bond donation (**D**) does the axial Fe-bound aqua complex remain 6-coordinate. Structure **D** also included hydrogen bond donation from the Fe-bound aqua ligand to the Tyr159 hydroxyl O-atom.

[*cis*-C3 (**3MPA**/NO)-MDO] coordinates

H	0.699314000000	2.740890000000	-5.747654000000
C	-0.252126000000	2.420002000000	-5.289641000000
C	-0.036634000000	1.466498000000	-4.166859000000
C	-0.179459000000	1.622972000000	-2.807708000000
N	0.396457000000	0.162401000000	-4.327995000000
C	0.494110000000	-0.416476000000	-3.106375000000
N	0.155382000000	0.447506000000	-2.166712000000
H	-0.875534000000	1.987828000000	-6.091049000000
H	-0.764376000000	3.318408000000	-4.917363000000
H	-0.506315000000	2.499688000000	-2.262200000000
H	0.595657000000	-0.294161000000	-5.213086000000
H	0.798936000000	-1.444199000000	-2.925109000000
H	-1.276236000000	6.208963000000	0.487101000000
C	-1.121158000000	5.516312000000	1.332434000000
C	-0.440501000000	4.278757000000	0.875789000000
C	-0.844603000000	2.964822000000	0.815184000000
N	0.836448000000	4.283740000000	0.346396000000
C	1.162501000000	3.019206000000	-0.010293000000
N	0.159973000000	2.202709000000	0.251826000000
H	-2.101826000000	5.292054000000	1.775462000000
H	-0.532714000000	6.053481000000	2.094864000000
H	-1.780732000000	2.521916000000	1.138379000000
H	1.437272000000	5.097157000000	0.253436000000
H	2.117186000000	2.742478000000	-0.445535000000
H	5.517055000000	1.234026000000	-2.149357000000
C	5.666776000000	0.776273000000	-1.161894000000
C	4.366372000000	0.381228000000	-0.551447000000
C	3.063926000000	0.512563000000	-0.976268000000
N	4.275732000000	-0.255358000000	0.672794000000
C	2.970711000000	-0.475210000000	0.954066000000
N	2.209982000000	-0.013890000000	-0.022347000000
H	6.215435000000	1.497789000000	-0.533462000000
H	6.312736000000	-0.107532000000	-1.296641000000
H	2.691756000000	0.946363000000	-1.898136000000
H	5.058242000000	-0.524756000000	1.262006000000
H	2.623880000000	-0.964582000000	1.859373000000
H	-5.270075000000	2.447549000000	7.685983000000
C	-5.053045000000	3.412735000000	7.200900000000
H	-5.558467000000	4.205996000000	7.774595000000
C	-5.493145000000	3.416295000000	5.728279000000
C	-4.789591000000	2.370815000000	4.888512000000
C	-3.454828000000	2.540641000000	4.486673000000
C	-5.428209000000	1.176292000000	4.516914000000
C	-2.776518000000	1.559975000000	3.757079000000
C	-4.768702000000	0.188382000000	3.783253000000
C	-3.430959000000	0.369065000000	3.403681000000
O	-2.825807000000	-0.634431000000	2.717120000000
H	-3.966384000000	3.581349000000	7.275491000000
H	-5.300242000000	4.416766000000	5.302599000000
H	-6.582519000000	3.257152000000	5.667903000000
H	-2.923866000000	3.459332000000	4.755384000000
H	-6.469866000000	1.013527000000	4.809561000000
H	-1.733588000000	1.713157000000	3.471245000000
H	-5.276108000000	-0.735774000000	3.497338000000
H	-1.912191000000	-0.400495000000	2.476313000000

H	-5.581140000000	8.795306000000	-4.602836000000
C	-4.515786000000	8.555154000000	-4.753731000000
H	-4.412601000000	8.110871000000	-5.757497000000
C	-3.958858000000	7.630848000000	-3.666110000000
C	-4.697743000000	6.299413000000	-3.513833000000
C	-4.035455000000	5.386706000000	-2.479817000000
N	-4.848201000000	4.201520000000	-2.234260000000
C	-4.423897000000	3.041557000000	-1.703678000000
N	-3.138003000000	2.870900000000	-1.364272000000
N	-5.273191000000	2.033664000000	-1.532296000000
H	-3.954950000000	9.501861000000	-4.745544000000
H	-3.976165000000	8.156928000000	-2.694740000000
H	-2.893447000000	7.427914000000	-3.879441000000
H	-4.746102000000	5.771721000000	-4.482312000000
H	-5.741951000000	6.493738000000	-3.207247000000
H	-3.871588000000	5.943627000000	-1.537795000000
H	-3.045710000000	5.068753000000	-2.847128000000
H	-5.851324000000	4.303009000000	-2.352151000000
H	-2.819876000000	1.975813000000	-0.969617000000
H	-2.520749000000	3.665431000000	-1.258315000000
H	-6.228180000000	2.106449000000	-1.860748000000
H	-4.937488000000	1.171343000000	-1.051424000000
Fe	0.000803000000	-0.001003000000	-0.002798000000
C	-3.041628000000	-0.557034000000	-0.311418000000
O	-2.028097000000	0.245326000000	-0.282076000000
C	-2.813322000000	-2.061890000000	-0.294207000000
O	-4.211373000000	-0.144241000000	-0.360100000000
C	-1.649099000000	-2.572488000000	-1.144193000000
S	-0.000573000000	-2.364238000000	-0.382559000000
H	-2.672206000000	-2.361091000000	0.757511000000
H	-1.782549000000	-3.651668000000	-1.311456000000
H	-1.661919000000	-2.090384000000	-2.135893000000
H	-3.756405000000	-2.517625000000	-0.632426000000
N	0.017304000000	-0.011278000000	1.897616000000
O	0.647100000000	0.462330000000	2.781120000000

[*trans*-C3 (**3MPA**/NO)-MDO] coordinates

H	0.386317000000	2.870756000000	-5.562959000000
C	-0.528391000000	2.299941000000	-5.292334000000
C	-0.287321000000	1.370574000000	-4.153178000000
C	-0.273557000000	1.578430000000	-2.792421000000
N	0.003099000000	0.028001000000	-4.306977000000
C	0.167914000000	-0.525313000000	-3.081540000000
N	0.010605000000	0.393255000000	-2.145328000000
H	-0.869356000000	1.761649000000	-6.199152000000
H	-1.306918000000	3.042708000000	-5.030871000000
H	-0.457570000000	2.496584000000	-2.247501000000
H	0.066023000000	-0.468203000000	-5.190949000000
H	0.373908000000	-1.576487000000	-2.900130000000
H	-1.259009000000	6.154876000000	0.400734000000
C	-0.819885000000	5.621283000000	1.271044000000
C	-0.185779000000	4.354881000000	0.833764000000
C	-0.607209000000	3.044722000000	0.811631000000
N	1.055693000000	4.341316000000	0.225088000000
C	1.344621000000	3.066851000000	-0.137311000000
N	0.350971000000	2.270107000000	0.197018000000
H	-1.634508000000	5.480521000000	2.007471000000
H	-0.067279000000	6.295303000000	1.727984000000
H	-1.525039000000	2.604247000000	1.186515000000
H	1.653005000000	5.150082000000	0.083083000000
H	2.264248000000	2.771118000000	-0.633969000000
H	5.441884000000	0.882013000000	-2.598139000000
C	5.620789000000	0.587192000000	-1.546991000000
C	4.377292000000	0.292635000000	-0.810657000000
C	3.052999000000	0.352123000000	-1.161453000000
N	4.389530000000	-0.140653000000	0.501390000000
C	3.115717000000	-0.316306000000	0.909880000000
N	2.288007000000	-0.018640000000	-0.076160000000
H	6.181853000000	1.408167000000	-1.051761000000
H	6.281038000000	-0.304438000000	-1.555851000000
H	2.604589000000	0.638045000000	-2.106495000000
H	5.219640000000	-0.307044000000	1.062845000000
H	2.830520000000	-0.654087000000	1.901809000000
H	-4.569476000000	2.883258000000	8.025701000000
C	-4.378934000000	3.826131000000	7.470582000000
H	-4.834117000000	4.656200000000	8.050558000000
C	-5.007104000000	3.784660000000	6.069380000000
C	-4.493485000000	2.673284000000	5.183491000000
C	-3.208058000000	2.748754000000	4.628259000000
C	-5.251930000000	1.524318000000	4.902623000000
C	-2.702085000000	1.722913000000	3.832361000000
C	-4.759680000000	0.489581000000	4.098922000000
C	-3.471254000000	0.584307000000	3.558593000000
O	-2.980209000000	-0.429562000000	2.791246000000
H	-3.280046000000	3.985365000000	7.461570000000
H	-4.828518000000	4.753739000000	5.570845000000
H	-6.100247000000	3.686725000000	6.174001000000
H	-2.580522000000	3.622818000000	4.828034000000
H	-6.259398000000	1.433188000000	5.319868000000
H	-1.682883000000	1.777927000000	3.458098000000
H	-5.365363000000	-0.395162000000	3.889565000000
H	-2.104298000000	-0.168810000000	2.469072000000

H	-5.530658000000	9.037876000000	-4.387545000000
C	-4.551702000000	8.585592000000	-4.652289000000
H	-4.674719000000	8.078040000000	-5.632737000000
C	-3.983116000000	7.653079000000	-3.579217000000
C	-4.780806000000	6.378274000000	-3.301765000000
C	-4.056538000000	5.475941000000	-2.300648000000
N	-4.841617000000	4.285140000000	-2.003682000000
C	-4.369800000000	3.121691000000	-1.524940000000
N	-3.064645000000	2.967580000000	-1.246162000000
N	-5.182751000000	2.089662000000	-1.339080000000
H	-3.828223000000	9.414129000000	-4.792817000000
H	-3.869605000000	8.214865000000	-2.634560000000
H	-2.959337000000	7.363519000000	-3.878943000000
H	-4.943535000000	5.816952000000	-4.238521000000
H	-5.780359000000	6.635348000000	-2.906203000000
H	-3.840999000000	6.040386000000	-1.373827000000
H	-3.088520000000	5.162819000000	-2.725411000000
H	-5.849738000000	4.362790000000	-2.092053000000
H	-2.731503000000	2.073514000000	-0.869675000000
H	-2.453905000000	3.767201000000	-1.141355000000
H	-6.145007000000	2.132659000000	-1.651404000000
H	-4.768619000000	1.196817000000	-0.979279000000
Fe	0.232398000000	-0.015396000000	0.115994000000
C	-2.866927000000	-0.535430000000	-0.219702000000
O	-1.880373000000	0.262921000000	-0.009863000000
C	-2.692546000000	-2.052612000000	-0.181029000000
O	-4.014045000000	-0.139343000000	-0.500369000000
C	-1.519115000000	-2.653892000000	0.579321000000
S	0.116858000000	-2.356624000000	-0.189982000000
H	-3.636909000000	-2.445783000000	0.228591000000
H	-1.658214000000	-3.744491000000	0.624013000000
H	-1.524160000000	-2.293193000000	1.617879000000
H	-2.659479000000	-2.378795000000	-1.235863000000
N	0.275819000000	0.113469000000	2.000200000000
O	0.079162000000	0.336964000000	3.141259000000

XII. References

1. Abronina, P. I.; Galkin, K. I.; Backinowsky, L. V.; Grachev, A. A., Synthesis of a derivative of a pentasaccharide repeating unit of the O-antigenic polysaccharide of the bacterium Klebsiella pneumoniae O3 as a benzoylated 2-methoxycarbonylethyl thioglycoside. *Russian Chemical Bulletin* **2009,** *58* (2), 457-467.

2. Nawrath, T.; Gerth, K.; Müller, R.; Schulz, S., The Biosynthesis of the Aroma Volatile 2-Methyltetrahydrothiophen-3-one in the Bacterium Chitinophaga Fx7914. *ChemBioChem* **2010,** *11* (13), 1914-1919.

3. Qiu, X.; Yang, X.; Zhang, Y.; Song, S.; Jiao, N., Efficient and practical synthesis of unsymmetrical disulfides via base-catalyzed aerobic oxidative dehydrogenative coupling of thiols. *Organic Chemistry Frontiers* **2019,** *6* (13), 2220-2225.

4. York, N. J.; Lockart, M. M.; Sardar, S.; Khadka, N.; Shi, W.; Stenkamp, R. E.; Zhang, J.; Kiser, P. D.; Pierce, B. S., Structure of 3-mercaptopropionic acid dioxygenase with a substrate analog reveals bidentate substrate binding at the iron center. *J. Biol. Chem.* **2021,** *296*.

5. Sardar, S.; Weitz, A.; Hendrich, M. P.; Pierce, B. S., Outer-Sphere Tyrosine 159 within the 3-Mercaptopropionic Acid Dioxygenase S-H-Y Motif Gates Substrate-Coordination Denticity at the Non-Heme Iron Active Site. *Biochemistry* **2019,** *58* (51), 5135-5150.

6. Pierce, B. S.; Subedi, B. P.; Sardar, S.; Crowell, J. K., The “Gln-Type” Thiol Dioxygenase from Azotobacter vinelandii Is a 3-Mercaptopropionic Acid Dioxygenase. *Biochemistry* **2015,** *54* (51), 7477-7490.
